# Maternal air pollution exposure and postpartum depression: a systematic review and meta-analysis

**DOI:** 10.7189/jogh.16.04020

**Published:** 2026-03-06

**Authors:** Chu Li, Yuyao Jin, Wanwan Xu, Yuqi Shao, Yingying Hu

**Affiliations:** 1 Department of Gynecology, The Fourth Affiliated Hospital of School of Medicine, and International School of Medicine, International Institutes of Medicine, Zhejiang University, Yiwu, China; 2 Department of Obstetrics, The Fourth Affiliated Hospital of School of Medicine, and International School of Medicine, International Institutes of Medicine, Zhejiang University, Yiwu, China

## Abstract

**Background:**

Air pollution is an environmental stimulus that may predispose pregnant women to postpartum depression (PPD). However, the relationship between maternal exposure to air pollutants and PPD is still unclear. Understanding the magnitude of this effect is critical to developing public health policies that protect women's reproductive health.

**Methods:**

We searched all studies published in PubMed, Embase, Scopus, and Web of Science up to December 2024. The research protocol has been registered in PROSPERO. Test for homogeneity based on Cochran’s Q and *I*^2^ statistics was calculated, and the restricted maximum likelihood random effect model was applied. We assessed the overall quality of pooled estimates, the influence of single studies on the meta-analytic estimates, sources of between-study heterogeneity, and publication bias.

**Results:**

Of the 7881 unique publications identified, nine studies met the inclusion criteria for final review, involving 405 635 pregnant women. We comprehensively assessed the available data on air pollutants and PPD risk. Maternal exposure to particulate matter diameter ≤10 μm (PM_10_) increases the risk of PPD (pooled odds ratio (OR) = 1.08; 95% CI = 1.02–1.14, the whole pregnancy; pooled OR = 1.09; 95% CI = 1.03–1.15, the second trimester). Additionally, PPD was significantly associated with an increase of carbon monoxide (CO), nitrate ion (NO_3_^-^), and ammonium ion (NH_4_^+^).

**Conclusions:**

Maternal exposure to PM_10_, CO, NO_3_^-^, and NH_4_^+^ during pregnancy is associated with PPD occurrence, especially in the second trimester. Interventions to improve air pollutants may mitigate the maternal risks of developing PPD. Our findings support public health interventions and environmental policy reforms to protect maternal mental health.

**Registration:**

PROSPERO CRD42024626359

Postpartum depression (PPD), a prevalent complication affecting 10.0–31.5% of mothers, typically emerges within the first six months postpartum [1–4]. The condition carries serious consequences for mothers and their offspring, including childhood conduct problems [5], strained marital relationships [6], an elevated maternal disability and suicide risk [7]. While established risk factors include antenatal depression and psychosocial stressors [7–10], emerging evidence implicates environmental triggers – particularly air pollution – in PPD pathogenesis.

According to the report of Global Burden of Disease (GBD) (2019), air pollution ranks among the top five global mortality risk factors with particulate matter (diameter ≤2.5 μm (PM_2.5_)) being the fourth leading risk factor for death worldwide [11]. Extensive research evidence supported the neuropsychiatric effects of air pollutants, including particulate matter (PM_2.5_ and diameter ≤10 μm (PM_10_)), gaseous pollutants (nitrogen dioxide (NO_2_), sulphur dioxide (SO_2_), carbon monoxide (CO), and ozone (O_3_)) [12–15], yet remains inconsistent across studies.

Currently, several studies present conflicting findings: while some studies report significant associations between gestational PM_2.5_, PM_10_, CO, NO_2_, and SO_2_ exposure and PPD risk [16,17], others show null effects [18]. These discrepancies likely reflect critical differences in study design, including variations in exposure assessment methods and timing. Notably, the potential for trimester-specific vulnerability warrants careful examination, as different stages of pregnancy may show varying sensitivity to environmental insults. Methodological challenges further complicate the interpretation of existing evidence. Studies vary substantially in their approaches to exposure quantification, ranging from simple residential proximity models to sophisticated personal monitoring systems. Similarly, heterogeneity in PPD assessment tools and diagnostic criteria across studies may obscure true associations. These limitations highlight the need for a rigorous synthesis of available evidence to clarify the nature and magnitude of any pollution-PPD relationship.

There was a systematic review that reported the correlation between air pollution exposure and depression in 2022, which found significant associations between depression and exposure to PM_2.5_, CO, NO_2_, and SO_2_ [19]. However, they excluded pregnant women or post-partum depression. A recent meta-analysis of three articles published before May 2022 also reported a significant relationship between air pollution and PPD risk [20], reporting that PM_10_ exposure significantly increased PPD risk.

This meta-analysis is the first to systematically evaluate how exposure timing (by trimester) modifies maternal health outcomes, addressing a critical problem in existing reviews. We appeal to the public to pay more attention to the impact of ambient air pollution on women’s reproductive health.

## METHODS

### Search strategy

This study was conducted based on the Preferred Reporting Items for Systematic Preferred Reporting Items for Systematic reviews and Meta-Analysis (PRISMA) guidelines (Table S1 in the **Online Supplementary Document**) [21]. The study protocol was registered with PROSPERO (CRD42024626359). We reviewed four databases (PubMed, Web of Science, Scopus, and Embase) for research up to 20 December 2024. Keywords related to ‘air pollution’ and ‘Depression, Postpartum’ were used in these databases (Table S2 in the **Online Supplementary Document**).

### Selection criteria

The criteria for inclusion of studies were established prior to the literature search. These criteria were as follows:

1) The studies included human beings as participants, and at least one air pollutant involved in a study as follows: PM_2.5_, PM_10_, CO, SO_2_, and O_3_;

2) Study outcomes referred to postpartum depression;

3) The outcomes were PPD clinically diagnosed or documented in medical records;

4) The literature should be peer-reviewed publication;

5) We restricted the languages of the literature to English.

### Data extraction

Two researchers (CL and YYJ) independently screened and extracted data from the retrieved articles according to the inclusion criteria. All potentially eligible publications were first identified by browsing through titles, abstracts, and keywords. In addition, the reference lists of the included articles were also perused for further potentially relevant literature. The full texts of selected articles were assessed to determine whether they met the criteria. Discrepancies in the evaluation process were resolved through discussion with another researcher (YQS).

The following data was extracted from eligible studies: author, publication year, study location, period, study design, sample size, types and concentrations of air pollutants, exposure windows, assessment methods, adjustment variables, diagnostic criteria, effect estimates (*e.g*. odds ratio (OR)) with 95% confidence intervals (CIs). For those studies that only reported results in the figures, we used Web Plot Digitizer software to extract the values from the graphs [22]. In addition, we contacted the authors to obtain specific effect estimates reporting incomplete data.

### Quality and risk of bias assessment

The Newcastle-Ottawa Quality Assessment Scale (NOS) was utilised to evaluate the quality of cohort, case-control, and longitudinal studies. Among them, cohort studies were classified into three quality levels: low (0–3), moderate (4–6), and high (7–9). There is no suitable scale to assess the quality of time-series research. Based on earlier systematic reviews [23–25], there was employed to evaluate the risk of bias according to the following aspects: validation of patient-reported outcome measures, the quality of air pollutant measurements, and the extent of confounder adjustment. A study was rated as low quality if any of the three items received a score of zero, whereas a score between one and four points indicated moderate quality (Table S3 in the **Online Supplementary Document**).

The NOS was prioritised for observational studies (cohort/ case-control) due to its validated assessment of selection bias and confounding control, while the National Institutes of Environmental Health Sciences National Toxicology Program Office of Health Assessment and Translation (OHAT) was applied to experimental and time-series studies given its superior evaluation of exposure characterisation and temporal relationships to assess the risk of bias (ROB) in each study [26,27]. The evaluation covered key criteria (exposure assessment, outcome assessment, and confounding bias) and additional criteria (selection bias, attrition/exclusion bias, selective reporting bias, conflict of interest, and other biases). Studies classified as ‘probably high’ or ‘high’ risk for the key criteria, or the majority of additional criteria, were excluded. The ROB assessment standards are detailed in Table S4 in the **Online Supplementary Document**.

### Statistical analysis

To harmonise the diverse effect estimates reported for air pollutant-PPD associations, we standardised all estimates to reflect a 10 μg/m^3^ increase in pollutant concentration. This threshold was selected based on three considerations:

1) alignment with the World Health Organization (WHO) Air Quality Guideline interim targets for PM_2.5_ and PM_10_, where 10 μg/m^3^ represents clinically meaningful exposure gradations [28];

2) consistency with recent high-impact cohort studies in environmental epidemiology [29,30]; and

3) practical interpretability for public health applications. Where necessary, ppb values were converted to μg/m^3^ using the following formula (at 1 atmospheric pressure and 25°C) [26,31].



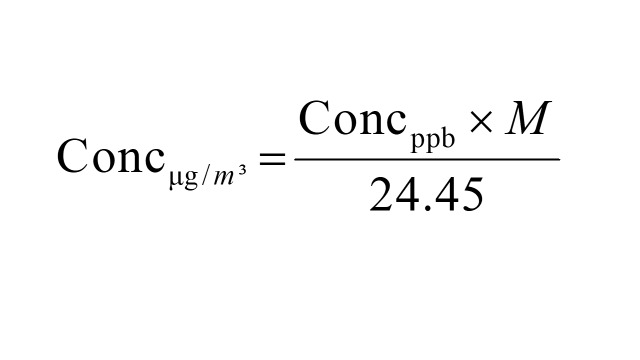



Concerning the choice of estimation method, we recognise that the random-effects model selection required more detailed rationale. Consistent with methodological recommendations for high-heterogeneity scenarios (*I*^2^>90%), we prioritised maximum likelihood random-effects (ML-RE) over DerSimonian-Laird (DL) estimators due to the latter's known downward bias in τ^2^ estimation under such conditions [32]. ML-RE demonstrated superior computational convergence compared to REML in simulation studies [33], to reduced bias in confidence interval coverage when dealing with extreme heterogeneity. In the present meta-analysis, the ML-RE was used to combine standardised effect estimates between air pollutants exposure and PPD. Heterogeneity between studies was assessed by Cochran's Q test and quantified using the statistic *I*^2^, where *I*^2^>50% was considered statistically significant heterogeneity [34]. Subgroup analyses were applied to explore potential sources of heterogeneity and effect modification of study characteristics. Studies were grouped according to exposure windows (pregnancy average, first trimester, second trimester, third trimester, postpartum 3–6 months), study regions, study designs (cohort and time-series study), sample size (<1000 and ≥1000), diagnostic criteria (centre for epidemiological studies-depression (CES-D), Edinburgh postnatal depression scale (EPDS), international classification of diseases, 9th and 10th (ICD 9/10) and other criteria [35]) and exposure evaluation methods (monitoring data (municipal or fixed-point monitoring), and model data (spatial-temporal interpolation or satellite data simulation)).

Sensitivity analysis was conducted by iteratively excluding individual studies to assess the robustness of the results. Publication bias was visualised via funnel plot and verified by Begg’s and Egger’s tests. All statistical analyses were performed using Stata 16 (StataCorp LLC, College Station, TX, USA).

## RESULTS

### Literature retrieval and study characteristics

**Figure 1:**
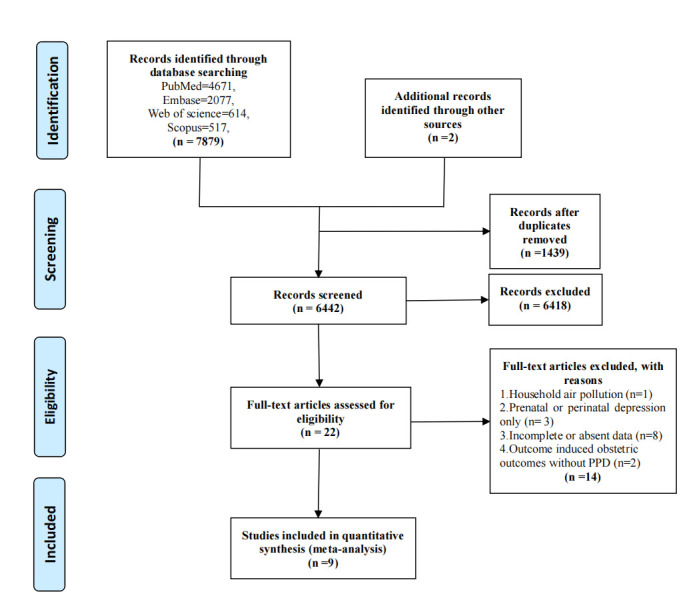
PRISMA flow diagram of the systematic review and meta-analysis.

There were 7879 relevant publications retrieved from four databases (PubMed = 4671, Embase = 2077, Web of Science = 614, and Scopus = 517). After examining all the references from full-text articles, two additional studies were identified. Of the total 7881 studies, 6442 publications after removing duplicates. Among the remaining 22 full-text articles, 13 articles were excluded as follows: study on household air pollution [36], and studies on prenatal or perinatal depression only [37–39]. Eight studies reported incomplete or absent data, such as no integral data for air pollution and PPD [40–47]. Two articles failed to make it into the finalist as they mainly focused on the relationship between air pollution exposure and obstetric outcomes during pregnancy without PPD [48,49]. Ultimately, nine original articles that met the criteria were included and subjected to systematic review and meta-analysis [16–18,50–55]. Details of the study selection process can be illustrated in **Figure 1**.

**Figure 2:**
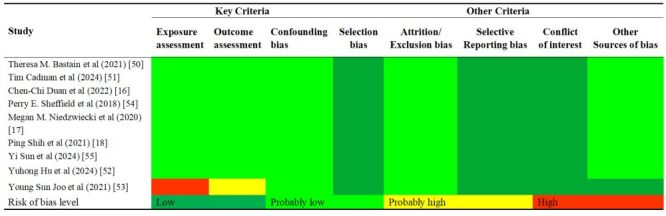
Risk of bias assessment for identified studies. According to the National Institutes of Environmental Health Sciences National Toxicology Program Office of Health Assessment and Translation (OHAT) tool and the University of California at San Francisco Navigation Guide, risk of bias assessment was carried out for every study. Each item was categorised as ‘low’, ‘probably low’, ‘probably high’, or ‘high’ risk on the basis of specific criteria. The OHAT tool suggested excluding studies for that the key criteria and most of the other criteria are characterised as ‘high’ or ‘probably high’ risk.

The characteristics of the studies satisfying the criterion are presented in **Table 1**. Among nine studies, seven were cohort studies [16–18,50,51,54,55] and two were time-series studies [52,53]. We divided all studies into three groups based on the original location of the study population: Asian studies were from China and Korea. Studies from the USA, Boston, Mexico, and Southern California were allocated to the American group. European studies contained reports from France, the UK, the Netherlands, Spain, Norway, Italy, and Greece. Since their inclusion and exclusion criteria are similar, we combine them to increase the sample size. The population sizes between different studies varied significantly, ranging from 180 to 340 679. There was considerable inconsistency in the diagnostic criteria used to diagnose PPD across studies. The outcome was defined using CES-D in two studies [50,52], EPDS in 4 studies [16,17,51,54], ICD code in 1 study [55], whereas two studies used self-administered questionnaires or scales [18,53]. PPD was assessed from one to 144 weeks, with this study analysing data at around six months postpartum. Six studies assigned participants pollutant exposure values based on monitoring station or self-monitoring data, while the remaining studies used models (spatio-temporal interpolation or satellite data simulation) to fit exposures. Six studies could be regarded as high quality [16–18,50,51,55], two moderate quality [52,54], and one poor quality [55]. ROB judgements for each included study are shown in **Figure 2**. None of the nine studies were excluded because of the high risk of bias.

**Table 1:** Characteristics of included studies

Author (year)	Country	Study years	Study design	Sample size	PPD number	Exposure period	Pollutants (unit)	Exposure assessment	Diagnostic criteria	Quality*
Theresa M. Bastain (2021) [50]	USA	2015–2020	Prospective cohort study	180	29	PA, T1, T2, T3	NO_2_(ppb), O_3_(ppb), PM_2.5_ (μg/m^3^), PM_10_ (μg/m^3^)	US EPA Air Quality System	CES-D	9 (NOS)
Tim Cadman (2024) [51]	European	1997–2016	Prospective cohort study	30 772	3078	PA	NO_2_ (μg/m^3^), PM_2.5_ (μg/m^3^), PM_10_ (μg/m^3^)	LUR models	EPDS	8 (NOS)
Chen-Chi Duan (2022) [16]	China	2019–2021	Prospective cohort study	10 209	2182	PA, T1, T2, T3	PM_2.5_ (μg/m^3^), PM_10_ (μg/m^3^), SO_2_ (μg/m^3^), CO (mg/m^3^), NO_2_ (μg/m^3^), O_3_ (μg/m^3^)	China National Environmental Monitoring Center	EPDS	8 (NOS)
Yuhong Hu (2024) [52]	USA	2015–2023	Time-series study	361	34	T1, T2, T3	NO_2_ (ppb), O_3_ (ppb), PM_2.5_ (μg/m^3^), PM_10_ (μg/m^3^)	US EPA Air Quality System	CES-D	4 [23]
Perry E Sheffield (2018) [54]	Boston	2002–2007	Prospective cohort study	346	47	PA	PM_2.5_ (μg/m^3^)	US EPA Air Quality System	EPDS	6 (NOS)
Megan M Niedzwiecki (2020) [17]	Mexico City	2007–2011	Prospective cohort study	509	90	PA, 0–12 mo PP	PM_2.5_ (μg/m^3^)	Hybrid satellite-based spatio-temporally resolved model	EPDS	9 (NOS)
Young Sun Joo (2021) [53]	Korea	2018	Time-series study	1391	27	PA	PM_2.5_ (μg/m^3^)	Average the worst levels of particles during 2018	Kessler [35]	3 [23]
Ping Shih (2021) [18]	China	2005	Prospective cohort study	21 188	3648	T1, T2, T3, 0–3 mo PP	PM_2.5_ (μg/m^3^), CO (ppm), NO_2_ (ppb)	Hybrid kriging/LUR and integrated. LUR-machine learning model based on data from the air monitoring stations	DSM-IV-TR>15	9 (NOS)
Yi Sun (2023) [55]	Southern California	2008–2016	Retrospective cohort study	340 679	25 674	PA, T1, T2, T3, 0–3 mo PP	PM_2.5_ (μg/m^3^), PM_10_ (μg/m^3^), NO_2_ (ppb), O_3_ (μg/m^3^), SO_4_^2−^ (ppb), NO_3_^−^ (ppb), NH_4_^+^ (ppb), BC (ppb)	Satellite, ground-based monitor, and chemical transport modelling data	ICD 9/10	9 (NOS)

EPA – environmental protection agency, LUR – land-use regression, PA – pregnancy average, PP – Postpartum, T1 – the first trimester, T2 – the second trimester, T3 – the third trimester

*The criteria of Mustafi´c et al., in 2012 can evaluate the quality of time-series, panel, and case-crossover studies. The full score is 5 of three dimensions which means high quality of literature. If the score of any dimension is 0 point, the quality of literature will be regard as low quality. Other situation is medium quality.

### Pooled estimates of ambient air pollution exposure on PPD risk

**Figure 3:**
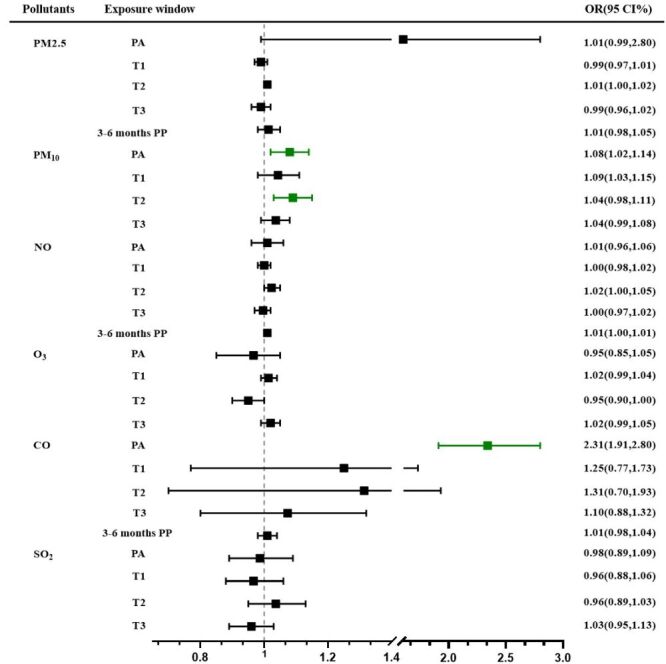
The summary of pooled OR and 95% CI on PPD associated with air pollutants. CI – confidence interval, OR – odds ratio, PA – pregnancy average, PP – postpartum, T1 – the first trimester, T2 – the second trimester, T3 – the third trimester.

The summary effect of the correlation between air pollution exposure and PPD risk is illustrated (**Figure 3**; Table S5 in the **Online Supplementary Document**).

For pollutants with multiple supporting studies, our meta-analysis demonstrated significant exposure-response relationships. PM_10_ exposure (n = 5) showed a consistent positive association with PPD risk, with each 10 μg/m^3^ increment during the entire pregnancy period corresponding to an adjusted OR of 1.08 (95% CI = 1.02–1.14, *I*^2^ = 90.2%, *P* < 0.001). This association was particularly pronounced during the second trimester (OR = 1.09; 95% CI = 1.03–1.15, *I*^2^ = 94.5%, *P* < 0.001 (Figure S1 in the **Online Supplementary Document**). However, primary analysis of PM_2.5_ exposure (n = 8) failed to demonstrate statistically significant associations across any gestational windows (Figure S2 in the **Online Supplementary Document**).

For single-study estimates, findings should be interpreted with appropriate caution due to the inability to assess heterogeneity or publication bias. Secondary analysis of PM_2.5_ constituents revealed that nitrate ion (NO_3_^-^) and ammonium ion (NH_4_^+^) components were significantly associated with PPD risk during mid-gestation (NO_3_^-^: OR = 1.02; 95% CI = 1.01–1.04; NH_4_^+^: OR = 1.02; 95% CI = 1.01–1.03). The reported association between CO exposure and PPD risk (OR = 2.31; 95% CI = 1.91–2.80 for whole-pregnancy exposure) (Figure S3 in the **Online Supplementary Document**) requires independent validation in future studies before meaningful clinical interpretation can be made.

### Meta-regression and heterogeneity exploration

The observed substantial heterogeneity (*I*^2 ^> 90%) prompted comprehensive sensitivity analyses to investigate potential modifying factors. We employed meta-regression models to quantitatively assess whether methodological or population-level characteristics could explain between-study variation. Different exposure windows, study regions, study design, sample size, diagnostic criteria, and exposure evaluation methods were further tested to look for potential causes of heterogeneity.

All analyses adhered to stringent methodological standards. Continuous covariates in meta-regression models met the minimum requirement of five studies per stratum to ensure stable estimation. For subgroups with fewer than three studies, we presented point estimates descriptively without pooling. For example, subgroup analysis by different sample types and exposure assessment methods to evaluate whether the conclusions from our study were sensitive to the restricted conditions of the included studies (Table S6–7 and Figure S4–5 in the **Online Supplementary Document**). Therefore, we consider these methods to be comparable across studies. However, it turned out that significant differences were observed only in the sample size, diagnostic criteria, and study design for PM_2.5_ (Table S6 in the **Online Supplementary Document**).

The complete analytical approach, including sensitivity analyses evaluating potential confounding and publication bias, is detailed in Figure S6–10 in the **Online Supplementary Document**. The results of the sensitivity analysis showed that the direction of each effect fitting value did not change after excluding each study one by one, indicating that our study was stable. Figure S11–15 show the details of the sensitivity analyses in the **Online Supplementary Document**. This multilayered approach provides confidence that the observed associations reflect true exposure-response relationships rather than methodological artifacts.

### Potential sensitive window

A total of three studies explored the potential sensitive window of air pollution exposure using distributed lag nonlinear models (DLNM). Two of which observe a relatively stronger association between PM_2.5_ exposure at weeks 10–27, and weeks 13–20, respectively [52,54], suggesting that the second trimester is a sensitive window period. However, no obvious evidence of a sensitive window period was found in another study [16]. For PM_10_, Hu et al. (2024) found that 12–28 gestational weeks is a sensitive window, which is not consistent with Duan et al. (2022). A significant association of NO_2_ exposure with PPD was observed from early to late pregnancy [16], while Hu et al. (2024) indicated the stronger positive correlation in the 13 to 29 gestational weeks. For SO_2_, 9–27 gestational weeks were confirmed as sensitive windows of PPD risk by Duan et al. (2022). For CO, Hu et al. (2024) showed that the sensitive window existed in the 4–32 weeks of gestation. In addition, they found slight positive associations between prenatal O_3_ exposures and persistent postpartum depression risk across gestations, but none of the weekly association was significant.

## DISCUSSION

In our systematic review and meta-analysis of nine cohort studies involving 405 635 pregnant women, we provided a comprehensive assessment of the available data on air pollutants and PPD risk. The results showed that exposure to PM_10_, CO, NO_3_^-^, and NH_4_^+^ can significantly increase the risk of PPD. In particular, CO exposure had the most remarkable effect on PPD risk, and the effect of PM_10_, NO_3_^-^ or NH_4_^+^ exposure was relatively modest. Our findings suggested that the second trimester might be the vulnerable window for the effect of air pollutant exposure on PPD risk. Significant heterogeneity was found between most air pollutant-outcome combinations. Univariate meta-regression analyses showed that the sample size, diagnostic criteria, and study design for PPD explained the heterogeneity between studies. Moreover, the sensitivity analysis supported the robustness of the pooled effect estimates. Therefore, interventions to improve air pollutants may mitigate the maternal risks of developing postpartum depression.

Compared to Borroni et al and Pourhoseini et al. results [19,20], the relationship between PM_10_ exposure and PPD risk was more significant. For single-study estimates, findings that CO, NO_3_^-^, and NH_4_^+^ exposure and PPD risk may be significant in our meta-analysis. The study showed that exposure to PM_10_ within the second trimester was associated with higher odds of occurrence of PPD (OR = 1.26; 95% CI = 1.15–1.37, n = 2), which is similar to us (OR = 1.09; 95% CI = 1.03–1.15, n = 4). However, we failed to find the relationship between O_3_ exposure throughout pregnancy (OR = 0.95; 95% CI = 0.90–1.00, n = 4), which is inconsistent with the study of Pourhoseini et al. (2024) (OR = 0.86; 95% CI = 0.75–0.99, n = 2), due to the inclusion of different literature. Moreover, the limited number of articles may give a biased perspective. Our current meta-analysis builds on these previous reviews, including a more comprehensive literature search and an increased sample size. As a result, we believe that our estimates are more accurate and comprehensive.

While air pollution studies focused on pregnant women and postpartum depression are still limited, our findings generally align with the broader body of research in non-pregnant adults, indicating that various components of air pollution may differentially affect mental health at other life stages, mainly in populations of older adults [56–59]. The meta-analysis has linked specific air pollutants, such as PM, NO_2_, SO_2_, CO, and O_3_, to increased risks of depressive symptoms or mental disorders, though studies focusing on pregnant women were not included [19]. Based on a large, population-based prospective study, Yang et al. considered the significant associations between PM_2.5_, NO_2_, NO, and depression [60], which was inconsistent with Zhang et al. [61] and our study. Some studies have frequently reported a positive correlation between ambient levels of SO_2_ and the manifestation of depressive symptoms [56,59]. Reversely, SO_2_ may demonstrate an inverse association with depressive symptoms or yield null results [62,63]. Other studies reported that O_3_ exposure was associated with depressive symptoms among middle-aged and older adults [59] as well as adolescents [64].

Mounting evidence suggests air pollution may contribute to depressive symptoms in pregnancy through distinct biological pathways. The inflammatory response appears particularly consequential, with trimester-specific patterns emerging. Exposure to PM_2.5_ during the second trimester (weeks 18–24) has been associated with elevated IL-6 levels and subsequent worsening of depressive symptoms [65], potentially mediated through pregnancy-enhanced indoleamine 2,3-dioxygenase activity that depletes central serotonin [66,67]. Air pollution's impact on the hypothalamic-pituitary-adrenal (HPA) axis demonstrates stage-dependent effects. Early pregnancy exposure may cause abnormal cortisol elevation that normally serves protective anti-inflammatory functions [68].

Unlike general depression, air pollution's effects during pregnancy appear uniquely tied to foetomaternal interface biology. PM_2.5_ may compromise the placenta's filtering capacity for inflammatory mediators, leading to abnormal release of proinflammatory cytokines into maternal circulation [69–73]. Where the original version drew parallels to general depression mechanisms, we now emphasise distinctive features of postpartum depression. The maternal neuroendocrine system undergoes dramatic changes during pregnancy, largely due to the endocrine effects of the developing placenta [74,75]. This differs fundamentally from chronic stress pathways in non-puerperal depression. Mor G et al. identified early-gestational mitochondrial vulnerability to oxidative stress and late-pregnancy immune-vascular crosstalk (possibly involving blood-brain barrier permeability) [75]. The advance seem could elucidate why identical pollutant concentrations exert differential effects across gestation stages. Future studies should examine mechanisms linking air pollution and other environmental exposures during pregnancy with postpartum psychological functioning.

The strength of this study is that we not only comprehensively searched all articles related to air pollution and PPD but also incorporated many new high-quality articles to assess the relationship between air pollutant exposure and PPD risk. Moreover, our meta-analysis included all the available recent studies to minimise potential selection bias. We also performed subgroup analysis to further explore the influence of various covariates on pooled estimates and performed meta-regression to explore sources of high heterogeneity.

However, we also noted some limitations in our study. First, while rigorous inclusion and exclusion criteria were implemented to enhance consistency, heterogeneity persisted across studies in terms of outcome measures, sample sizes, diagnostic criteria for PPD, and adjusted covariates, possibly affecting the validity of our estimates. Second, geographic representation biases – notably the underrepresentation of low- and middle-income countries – may limit the generalisability of findings, while residual confounding factors could persist even after statistical adjustments. In addition, the dose-response relationships were indeed not examined and in our current study, due to limited variability in exposure levels and data missing across the included studies. Regarding multi-pollutant models, we acknowledge this as an important limitation, as most original studies focused on single-pollutant analyses and incomplete raw data. These aspects represent valuable directions for future research when more comprehensive data become available.

## CONCLUSIONS

In summary, our findings demonstrate the association between air pollution (*e.g*. PM_10_, CO, NO_3_^-^, and NH_4_^+^) and PPD, particularly during the second trimester. Given the modifiable nature of air pollution, we urge policymakers to integrate trimester-specific air quality monitoring into prenatal care programmes, alongside public health campaigns to reduce maternal exposure during critical pregnancy windows. Air pollution alert systems should be established for prenatal care providers. Further large-scale cohort studies and mechanistic research are needed to establish causality and elucidate underlying biological pathways.

## Additional material


Online Supplementary Document
