# Supplementary material for: Maternal air pollution exposure and postpartum depression: a systematic review and meta-analysis
Source: J Glob Health. 2026 Mar 6;16:04020. doi: 10.7189/jogh.16.04020 (PMC12968735; doi:10.7189/jogh.16.04020)
Supplement: Online Supplementary Document [file jogh-16-04020-s001.pdf]

Supplement to: Li C, Jin Y, Xu W, Shao Y, Hu Y. Maternal air pollution exposure and postpartum depression: A systematic review and meta-analysis. J Glob Health. 2026;16:04020.

Catalogue

Table

Table S1	PRISMA 2020 item checklist
Table S2	The detail of search strategies
Table S3	Criteria of a scale developed to assess the quality of studies
Table S4	Criteria for the risk of bias assessment of each study, adapted from the OHAT and UCSF Navigation Guide tool
Table S5	The summary of pooled OR and 95%CI on PPD associated with air pollutants.
Table S6	Stratified analysis of exposure to particulate matter and PPD.
Table S7	The meta-regression to assess publication bias.

Figure

Figure S1	Meta-analysis of PM_{10} exposure and the risk of PPD.
Figure S2	Meta-analysis of $PM_{2.5}$ exposure and the risk of PPD.
Figure S3	Meta-analysis of CO exposure and the risk of PPD.
Figure S4	Stratified analysis of defined (CES-D, EPDS, ICD, etc.) with PPD.
Figure S5	Stratified analysis of exposure assessment methods (e.g., satellite, models) with PPD.
Figure S6	Funnel plots of the combined associations of $PM_{2.5}$ with PPD.
Figure S7	Funnel plots of the combined associations of PM_{10} with PPD.
Figure S8	Funnel plots of the combined associations of NO_2 with PPD.
Figure S9	Funnel plots of the combined associations of O_3 with PPD.
Figure S10	Funnel plots of the combined associations of CO with PPD.
Figure S11	Leave-one-out sensitivity analysis to assess the association between $PM_{2.5}$ and PPD.
Figure S12	Leave-one-out sensitivity analysis to assess the association between PM_{10} and PPD.
Figure S13	Leave-one-out sensitivity analysis to assess the association between NO_2 and PPD.
Figure S14	Leave-one-out sensitivity analysis to assess the association between O_3 and PPD.
Figure S15	Leave-one-out sensitivity analysis to assess the association between CO and PPD.

Table S1 PRISMA 2020 item checklist

Section/topic	#	Checklist item	Reported on page #
**TITLE**			
Title	1	Identify the report as a systematic review, meta-analysis, or both.	1
**ABSTRACT**			
Structured summary	2	Provide a structured summary including, as applicable: background; objectives; data sources; study eligibility criteria, participants, and interventions; study appraisal and synthesis methods; results; limitations; conclusions and implications of key findings; systematic review registration number.	2
**INTRODUCTION**			
Rationale	3	Describe the rationale for the review in the context of what is already known.	3
Objectives	4	Provide an explicit statement of questions being addressed with reference to participants, interventions, comparisons, outcomes, and study design (PICOS).	3-4
**METHODS**			
Protocol and registration	5	Indicate if a review protocol exists, if and where it can be accessed (e.g., Web address), and, if available, provide registration information including registration number.	4
Eligibility criteria	6	Specify study characteristics (e.g., PICOS, length of follow-up) and report characteristics (e.g., years considered, language, publication status) used as criteria for eligibility, giving rationale.	4<br>Table 1
Information sources	7	Describe all information sources (e.g., databases with dates of coverage, contact with study authors to identify additional studies) in the search and date last searched.	4
Search	8	Present full electronic search strategy for at least one database, including any limits used, such that it could be repeated.	4<br>Table S2
Study selection	9	State the process for selecting studies (i.e., screening, eligibility, included in systematic review, and, if applicable, included in the meta-analysis).	4<br>Figure 1
Data collection process	10	Describe method of data extraction from reports (e.g., piloted forms, independently, in duplicate) and any processes for obtaining and confirming data from investigators.	4-5
Data items	11	List and define all variables for which data were sought (e.g., PICOS, funding sources) and any assumptions and simplifications made.	4
Risk of bias in individual studies	12	Describe methods used for assessing risk of bias of individual studies (including specification of whether this was done at the study or outcome level), and how this information is to be used in any data synthesis.	5<br>Table S3-S5
Summary measures	13	State the principal summary measures (e.g., risk ratio, difference in means).	5
Synthesis of results	14	Describe the methods of handling data and combining results of studies, if done, including measures of consistency (e.g., I^2) for each meta-analysis.	5

Risk of bias across studies	15	Specify any assessment of risk of bias that may affect the cumulative evidence (e.g., publication bias, selective reporting within studies).	5<br>Table S3-S5
Additional analyses	16	Describe methods of additional analyses (e.g., sensitivity or subgroup analyses, meta-regression), if done, indicating which were pre-specified.	5,6
**RESULTS**			
Study selection	17	Give numbers of studies screened, assessed for eligibility, and included in the review, with reasons for exclusions at each stage, ideally with a flow diagram.	7<br>Figure 1
Study characteristics	18	For each study, present characteristics for which data were extracted (e.g., study size, PICOS, follow-up period) and provide the citations.	7-8<br>Table 1
Risk of bias within studies	19	Present data on risk of bias of each study and, if available, any outcome level assessment (see item 12).	11,13<br>Table S3-S5
Results of individual studies	20	For all outcomes considered (benefits or harms), present, for each study: (a) simple summary data for each intervention group (b) effect estimates and confidence intervals, ideally with a forest plot.	12-15<br>Figure 2-3, Figure S3-5
Synthesis of results	21	Present results of each meta-analysis done, including confidence intervals and measures of consistency.	12<br>Figure 2
Risk of bias across studies	22	Present results of any assessment of risk of bias across studies (see Item 15).	13<br>Table S3-S5
Additional analysis	23	Give results of additional analyses, if done (e.g., sensitivity or subgroup analyses, meta-regression [see Item 16]).	13-14<br>Table S6-S7, Figure S7-S11
**DISCUSSION**			
Summary of evidence	24	Summarize the main findings including the strength of evidence for each main outcome; consider their relevance to key groups (e.g., healthcare providers, users, and policy makers).	14-16
Limitations	25	Discuss limitations at study and outcome level (e.g., risk of bias), and at review-level (e.g., incomplete retrieval of identified research, reporting bias).	16
Conclusions	26	Provide a general interpretation of the results in the context of other evidence, and implications for future research.	16-17
**FUNDING**			
Funding	27	Describe sources of funding for the systematic review and other support (e.g., supply of data); role of funders for the systematic review.	17

Table S2. The detail of search strategies

Database	Search strategies	Results
Pubmed	(("Depression, Postpartum"[Mesh]) OR ("postnatal depression") OR ("postpartum depression") OR ("postpartum blahs") OR ("maternity blues") OR ("postpartum depress*") OR ("puerperal depressive symptom") OR ("after birth depression") OR ("prenatal depression") OR ("depression after childbirth")) AND (("Air Pollution"[Mesh]) OR (air contamination) OR (air pollutants) OR ("Particulate Matter"[Mesh]) OR (fine particulate matter) OR (PM2.5) OR (particulate matter 2.5) OR (inhalable particle) OR (inhalable particles) OR (PM10) OR (particulate matter 10) OR (inhalable particulate) OR (inhalable particulates) OR (ultra-fine particles) OR (ultrafine particles) OR (ultra-fine particle) OR (ultrafine particle) OR (ultrafine particulates) OR (ultrafine particulate) OR (ultra-fine particulate) OR(ultra-fine particulates) OR (PM0.1) OR (particulate matter 0.1) OR ("Sulfur Dioxide"[Mesh]) OR (SO2) OR ("Carbon Monoxide"[Mesh]) OR (CO) OR ("Nitrogen Dioxide"[Mesh]) OR (NOX) OR (NO2) OR ("Ozone"[Mesh]) OR ("Soot"[Mesh]) OR (black carbon) OR (BC) OR (PM light absorbance) OR (PM light absorption) OR (PM organic carbon) OR (PM elemental carbon) OR (PM10 light absorbance) OR (PM10 light absorption) OR (PM10 organic carbon) OR (PM10 elemental carbon) OR (PM2.5 light absorbance) OR (PM2.5 light absorption) OR (PM2.5 organic carbon) OR (PM2.5 elemental carbon) OR (PM0.1 light absorbance) OR (PM0.1 light absorption) OR (PM0.1 organic carbon) OR (PM0.1 elemental carbon))	4671
Embase	((‘Depression, Postpartum’/exp) OR (‘postnatal depression’) OR (‘postpartum depression’) OR (‘postpartum blahs’) OR (‘maternity blues’) OR (‘postpartum depress*’) OR (‘puerperal depressive symptom’) OR (‘after birth depression’) OR (‘prenatal depression’) OR (‘depression after childbirth’)) AND ((‘air pollution’/exp) OR (air contamination) OR (air pollutants) OR (‘particulate matter’/exp) OR (fine particulate matter) OR (PM2.5) OR (particulate matter 2.5) OR (inhalable particle) OR (inhalable particles) OR (PM10) OR (particulate matter 10) OR (inhalable particulate) OR (inhalable particulates) OR (‘ultrafine particulate matter’/exp) OR (ultra-fine particles) OR (ultrafine particles) OR (ultra-fine particle) OR (ultrafine particle) OR (ultrafine particulates) OR (ultrafine particulate) OR (ultra-fine particulate) OR (ultra-fine particulates) OR (PM0.1) OR (particulate matter 0.1) OR (‘sulfur dioxide’/exp) OR (SO2) OR (‘carbon monoxide’/exp) OR (CO) OR (‘nitrogen dioxide’/exp) OR (NOX) OR (NO2) OR (‘ozone’/exp) OR (‘black carbon’/exp) OR (soot) OR (BC) OR (PM light absorbance) OR (PM light absorption) OR (PM organic carbon) OR (PM elemental carbon) OR (PM10 light absorbance) OR (PM10 light absorption) OR (PM10 organic carbon) OR (PM10 elemental carbon) OR (PM2.5 light absorbance) OR (PM2.5 light absorption) OR (PM2.5 organic carbon) OR (PM2.5 elemental carbon) OR (PM0.1 light absorbance) OR (PM0.1 light absorption) OR (PM0.1 organic carbon) OR (PM0.1 elemental carbon))	2077
Scopus	(ALL("Depression, Postpartum") OR TITLE-ABS-KEY("postnatal depression") OR TITLE-ABS-KEY("postpartum depression") OR TITLE-ABS-KEY("postpartum blahs") OR TITLE-ABS-KEY("maternity blues") OR TITLE-ABS-KEY("postpartum depress*") OR TITLE-ABS-KEY("puerperal depressive symptom") OR TITLE-ABS-KEY("after birth depression") OR TITLE-ABS-KEY("prenatal depression") OR TITLE-ABS-KEY("depression after childbirth")) AND (ALL("air pollution") OR TITLE-ABS-KEY("air contamination") OR TITLE-ABS-KEY("air pollutants") OR ALL("particulate matter") OR TITLE-ABS-KEY("fine particulate matter") OR TITLE-ABS-KEY(PM2.5) OR TITLE-ABS-KEY("particulate matter 2.5") OR TITLE-ABS-KEY(inhalable particle) OR TITLE-ABS-KEY(inhalable particles) OR TITLE-ABS-KEY(PM10) OR TITLE-ABS-KEY(particulate matter 10) OR	517

	TITLE-ABS-KEY(inhalable particulate) OR TITLE-ABS-KEY(inhalable particulates) OR ALL("ultrafine particulate matter") OR TITLE-ABS-KEY(ultra-fine particles) OR TITLE-ABS-KEY(ultrafine particles) OR TITLE-ABS-KEY(ultra-fine particle) OR TITLE-ABS-KEY(ultrafine particle) OR TITLE-ABS-KEY(ultrafine particulates) OR TITLE-ABS-KEY(ultrafine particulate) OR TITLE-ABS-KEY(ultra-fine particulate) OR TITLE-ABS-KEY(ultra-fine particulates) OR TITLE-ABS-KEY(PM0.1) OR TITLE-ABS-KEY(particulate matter 0.1) OR ALL("sulfur dioxide") OR TITLE-ABS-KEY(SO2) OR ALL("carbon monoxide") OR TITLE-ABS-KEY(CO) OR ALL("nitrogen dioxide") OR TITLE-ABS-KEY(NOX) OR TITLE-ABS-KEY(NO2) OR ALL("ozone") OR ALL("black carbon") OR TITLE-ABS-KEY(soot) OR TITLE-ABS-KEY(BC) OR TITLE-ABS-KEY(PM light absorbance) OR TITLE-ABS-KEY(PM light absorption) OR TITLE-ABS-KEY(PM organic carbon) OR TITLE-ABS-KEY(PM elemental carbon) OR TITLE-ABS-KEY(PM10 light absorbance) OR TITLE-ABS-KEY(PM10 light absorption) OR TITLE-ABS-KEY(PM10 organic carbon) OR TITLE-ABS-KEY(PM10 elemental carbon) OR TITLE-ABS-KEY(PM2.5 light absorbance) OR TITLE-ABS-KEY(PM2.5 light absorption) OR TITLE-ABS-KEY(PM2.5 organic carbon) OR TITLE-ABS-KEY(PM2.5 elemental carbon) OR TITLE-ABS-KEY(PM0.1 light absorbance) OR TITLE-ABS-KEY(PM0.1 light absorption) OR TITLE-ABS-KEY(PM0.1 organic carbon) OR TITLE-ABS-KEY(PM0.1 elemental carbon))	
Web of science	(TS=(Depression, Postpartum) OR (postnatal depression) OR (postpartum depression) OR (postpartum blahs) OR (maternity blues) OR (postpartum depress*) OR (puerperal depressive symptom) OR (after birth depression) OR (prenatal depression) OR (depression after childbirth)) AND (TS=(Air Pollution) OR (air contamination) OR (air pollutants) OR (Particulate Matter) OR (fine particulate matter) OR (PM2.5) OR (particulate matter 2.5) OR (inhalable particle) OR (inhalable particles) OR (PM10) OR (particulate matter 10) OR (inhalable particulate) OR (inhalable particulates) OR (ultra-fine particles) OR (ultrafine particles) OR (ultra-fine particle) OR (ultrafine particle) OR (ultrafine particulates) OR (ultrafine particulate) OR (ultra-fine particulate) OR(ultra-fine particulates) OR (PM0.1) OR (particulate matter 0.1) OR (Sulfur Dioxide) OR (SO2) OR (Carbon Monoxide) OR (CO) OR (Nitrogen Dioxide) OR (NOX) OR (NO2) OR (Ozone) OR (black carbon) OR (BC) OR (soot) OR (PM light absorbance) OR (PM light absorption) OR (PM organic carbon) OR (PM elemental carbon) OR (PM10 light absorbance) OR (PM10 light absorption) OR (PM10 organic carbon) OR (PM10 elemental carbon) OR (PM2.5 light absorbance) OR (PM2.5 light absorption) OR (PM2.5 organic carbon) OR (PM2.5 elemental carbon) OR (PM0.1 light absorbance) OR (PM0.1 light absorption) OR (PM0.1 organic carbon) OR (PM0.1 elemental carbon))	614

Table S3. Quality assessment (NOS) of cohort studies

Included studies	Selection				Comparability	Exposure			Score
	①	②	③	④	⑤	⑥	⑦	⑧	
Theresa M. Bastain et al (2021)	★	★	★	★	★★	★	★	★	9
Tim Cadman et al (2024)	★	★	★	★	★	★	★	★	8
Chen-Chi Duan et al (2022)	★	★	★	★	★★	★		★	8
Perry E. Sheffield et al (2018)		★	★		★	★	★	★	6
Megan M. Niedzwiecki et al (2020)	★	★	★	★	★★	★	★	★	9
Ping Shih et al (2021)	★	★	★	★	★	★	★	★	8
Yi Sun et al (2024)	★	★	★	★	★★	★	★	★	9

Selection: ① Representativeness of the exposed cohort; ② Selection of the non-exposed cohort; ③Ascertainment of exposure; ④ Demonstration that outcome of interest was not present at the start of study;

Comparability: ⑤ Comparability of cohorts based on the design or analysis;

Outcome: ⑥ Assessment of outcome; ⑦ Was follow-up long enough for outcomes to occur; ⑧ Adequacy of follow up of cohorts

Criteria of a scale developed to assess the quality of time-series study by Mustafić et al. in 2012

**Item**		**Score**
Validation of patient-reported outcome measures	The absence of valid criteria	0
	the presence of valid criteria	1
The quality of air pollutant measurements	The measurements were not performed at least daily or that more than 25% of the data were missing	0
	The measurements were performed at least daily and that missing data were less than 25%	1
The extent of confounder adjustments	No adjustment was made for long-term trends, season, and air temperature	0
	Only long-term trends, season, and air temperature were adjusted	1
	Additional adjustment was made, including humidity or day of the week, in addition to the corresponding adjustments with the score of "1"	2
	Adjustment was made for holidays in addition to the corresponding adjustments with a score of "2"	3

Mustafic H, Jabre P, Caussin C, Murad MH, Escolano S, Tafflet M, Périer MC, Marijon E, Vernerey D, Empana JP, Jouven X. Main air pollutants and myocardial infarction: a systematic review and meta-analysis. JAMA. 2012 Feb 15; 307(7): 713-21.

Table S4. Criteria for the risk of bias assessment of each study, adapted from the OHAT and UCSF Navigation Guide tool

**Bias**		**Risk of Bias Domains and Ratings**	**Answer**
**Key Criteria**	**Detection bias, exposure assessment**	Can we be confident in the exposure characterization?<br>List of major considerations:<br>1) model was supplemented by representative measurements, traffic data and other geographic predictors of air pollution are available;<br>2) exact residential addresses are used to predict air pollution levels;<br>3) spatial resolution information of models was provided;<br>4) air pollutants were monitored by air monitoring stations daily and missing data were < 25%.	-LOW risk: There is high confidence that the exposure to air pollutions is the true average population exposure, or satisfies four listed major considerations.<br>-PROBABLY LOW: There is indirect evidence that suggests low risk of bias, or use models to predict air pollutions but two of the four listed considerations is not applied.<br>-PROBABLY HIGH risk: There is insufficient information to permit a judgment of high risk of bias, but there is indirect evidence that suggests high risk of bias. Only use monitoring stations as population exposure to air pollutions levels, and satisfies the last listed consideration.<br>-HIGH risk: There is direct evidence of high risk of misclassification bias, or all four of the listed considerations are not applied.
	**Detection bias, outcome assessment**	Can we be confident in the outcome assessment?	-LOW risk: Outcome was classified based on diagnosis standard criteria (International Classification System code) and provided by a national or regional database.<br>-PROBABLY LOW: Outcome was assessed based on diagnosis standard criteria and collected by researcher.<br>-PROBABLY HIGH risk: Outcome was not assessed based on standard diagnosis criteria AND is accompanied by validation sub-study or sensitivity analysis to suggest that the risk is minimum.<br>-HIGH risk: Outcome was assessed based on self-reports (parents, family) and data collected by the researcher.

	**Confounding bias**	Did the study design or analysis account for important confounding and modifying variables?<br>The potential confounder covariates selected using DAG including meteorological factors (e.g. temperature, relatively humidity, barometric pressure, sunlight hours, and wind speed), age, sex, ethnicity, household income, smoking, physical activity, day of week, season, urbanity, population density, region, occupation, domestic fuel type and ventilation, social-economic status, and the spent outside and time spent in front of a screen.<br>The potential mediators including TG, health problem, social satisfaction, sleep difficulties, and SBP.	-LOW risk: Study accounted for 4/5 of the DAG selected confounders (in our review, this kind of studies did not adjust for any potential mediator, thus we did not consider mediator in this item).<br>-PROBABLY LOW risk: Study accounted for 3/5 of the DAG selected confounders, and 1/5 of the potential mediators.<br>-PROBABLY HIGH risk: Study accounted for 2/5 of the DAG selected confounders and 2/5 of the selected mediators.<br>-HIGH risk: Study accounted for ≤ 1/5 DAG selected confounders and ≥ 3/5 of the selected mediators OR inappropriately over-adjusted four or more unrelated variables.
**Other Criteria**	**Selection bias**	Did selection of study participants result in appropriate comparison groups?	-LOW risk: The descriptions of the studied population were sufficiently detailed to support the assertion that risk of selection effects was minimal.<br>-PROBABLY LOW risk: There is insufficient information about population selection to permit a judgment of low risk of bias, but there is indirect evidence that suggests low risk of bias.<br>-PROBABLY HIGH risk: There is insufficient information about population selection to permit a judgment of high risk of bias, but there is indirect evidence that suggests high risk of bias.<br>-HIGH risk: There were indications from descriptions of the studied population of high risk of bias.

	**Attrition/exclusion bias**	Were outcome data complete without attrition or exclusion from analysis?	-LOW risk: There were no missing outcome data or missing data unrelated to true outcome.<br>-PROBABLY LOW: There was insufficient information about incomplete data to judge for low risk, but indirect evidence that suggests low risk of bias.<br>-PROBABLY HIGH risk: There was insufficient information about incomplete data to judge for high risk, but indirect evidence that suggests high risk.<br>-HIGH risk: Missing outcome data is related to true outcome.
	**Selective reporting bias**	Were all measured outcomes reported?	-LOW risk: All of the studies pre-specified outcomes and findings are reported.<br>-PROBABLY LOW: There was insufficient information about selective outcome to judge for low risk, but indirect evidence that suggests study was free of selective report.<br>-PROBABLY HIGH risk: There was insufficient information about selective reporting to judge for high risk, but indirect evidence suggests that study was not free of selective reporting.<br>-HIGH risk: Not all pre-specified outcomes and findings were reported, or one/more of the primary outcomes or analyses were assessed or executed with other methods than the pre-specified one, or one/more of the reported outcomes/findings was/were not pre-specified.
	**Conflict of interest**	Potential source of bias in reporting through source of funding	-LOW risk: The study did not receive funding from an entity with financial interest in the outcome of study.<br>-PROBABLY LOW: There is insufficient information to judge for low risk, but indirect evidence suggests study was free of financial interest.<br>-PROBABLY HIGH risk: There is insufficient information to judge for high risk, but indirect evidence suggests study was not free of financial interest.<br>-HIGH risk: The study received support from an entity with financial interest in the

			outcome of study.
	**Other source of bias**	Bias due to other problems not covered elsewhere (statistical methods were appropriate and researchers adhere to the study protocol)	-LOW risk: No other sources of bias.<br>-PROBABLY LOW: There is insufficient information to judge for low risk, but indirect evidence suggests study was free of other problems.<br>-PROBABLY HIGH risk: There is insufficient information to judge for high risk, but indirect evidence suggests study was not free of other problems.<br>-HIGH risk: There was at least one important risk of bias.

Fan SJ, Heinrich J, Bloom MS, Zhao TY, Shi TX, Feng WR, Sun Y, Shen JC, Yang ZC, Yang BY, Dong GH. Ambient air pollution and depression: A systematic review with meta-analysis up to 2019. Sci Total Environ. 2020 Jan 20;701:13472

Table S5. The summary of pooled OR and 95%CI on PPD associated with air pollutants.

Pollutants	Exposure window	Number of studies	Pooled OR	95%CI		Heterogeneity		P_{-Begg}	P_{-Egger}
						I^2(%)	*p* value		
$PM_{2.5}$	Pregnancy average	8	1.01	0.99	2.80	78.0	<0.001	1.000	0.286
	First trimester	6	0.99	0.97	1.01	34.7	0.176	0.573	0.504
	Second trimester	6	1.01	1.00	1.02	0.0	0.435	0.851	0.872
	Third trimester	6	0.99	0.96	1.02	70.6	0.004	0.851	0.726
	3-6 months PP	1	1.01	0.98	1.05	——	——	——	——
PM_{10}	Pregnancy average	5	**1.08**	**1.02**	**1.14**	90.2	<0.001	0.142	0.172
	First trimester	4	1.04	0.98	1.11	92.9	<0.001	1.000	0.517
	Second trimester	4	**1.09**	**1.03**	**1.15**	94.5	<0.001	1.000	0.538
	Third trimester	4	1.04	0.99	1.08	87.9	<0.001	1.000	0.443
NO_2	Pregnancy average	5	1.01	0.96	1.06	83.1	<0.001	0.327	0.552
	First trimester	5	1.00	0.98	1.02	89.0	<0.001	1.000	0.432
	Second trimester	5	1.02	1.00	1.05	93.1	<0.001	1.000	0.699
	Third trimester	5	1.00	0.97	1.02	83.7	<0.001	0.624	0.833
	3-6 months PP	1	1.01	1.00	1.01	——	——	——	——
O_3	Pregnancy average	4	0.95	0.85	1.05	95.2	<0.001	1.000	0.905
	First trimester	4	1.02	0.99	1.04	72.6	0.012	0.497	0.681
	Second trimester	4	0.95	0.90	1.00	94.8	<0.001	0.497	0.424
	Third trimester	4	1.02	0.99	1.05	73.1	0.011	0.497	0.747
CO	Pregnancy average	1	**2.31**	**1.91**	**2.80**	——	——	——	——
	First trimester	2	1.25	0.77	1.73	95.6	<0.001	0.317	——
	Second trimester	2	1.31	0.70	1.93	97.1	<0.001	0.317	——
	Third trimester	2	1.10	0.88	1.32	83.3	0.014	0.317	——
	3-6 months PP	1	1.01	0.98	1.04	——	——	——	——
SO_2	Pregnancy average	1	0.98	0.89	1.09	——	——	——	——
	First trimester	1	0.96	0.88	1.06	——	——	——	——
	Second trimester	1	1.03	0.95	1.13	——	——	——	——
	Third trimester	1	0.96	0.89	1.03	——	——	——	——
$PM_{2.5}$ BC	Pregnancy average	1	1.04	1.00	1.09	——	——	——	——
	First trimester	1	1.03	1.00	1.05	——	——	——	——
	Second trimester	1	0.99	0.97	1.03	——	——	——	——
	Third trimester	1	1.01	0.99	1.05	——	——	——	——
$PM_{2.5}$ SO_4^{2-}	Pregnancy average	1	1.01	1.00	1.03	——	——	——	——
	First trimester	1	0.99	0.99	1.00	——	——	——	——
	Second trimester	1	1.00	0.99	1.01	——	——	——	——
	Third trimester	1	1.01	1.00	1.02	——	——	——	——
$PM_{2.5}$ NO_3^-	Pregnancy average	1	1.01	0.99	1.04	——	——	——	——
	First trimester	1	0.99	0.97	1.00	——	——	——	——
	Second trimester	1	**1.02**	**1.01**	**1.04**	——	——	——	——
	Third trimester	1	1.00	0.99	1.02	——	——	——	——
$PM_{2.5}$ NH_4^+	Pregnancy average	1	1.02	0.99	1.04	——	——	——	——
	First trimester	1	0.99	0.97	1.00	——	——	——	——

Second trimester	1	**1.02**	**1.01**	**1.03**	——	——	——	——
Third trimester	1	1.01	0.99	1.02	——	——	——	——

PPD - postpartum depression, BC - black carbon, CI – confidence interval, PP - Postpartum

Table S6. Stratified analysis of exposure to air pollutants and PPD.

Pollutants	Exposure window	Subgroup	Division	Pooled OR	95%CI		Heterogeneity	
							I^2 (%)	p value
$PM_{2.5}$	Whole pregnancy	Region	America (n=5)	1.00	0.98	1.03	75.5	0.003
			Asia (n=2)	1.02	1.01	1.03	0.0	0.801
			Europe(n=1)	1.05	0.96	1.14	——	——
		Study design	Cohort study (n=7)	1.02	1.01	1.03	0.6	0.419
			Time-series study (n=1)	0.99	0.99	1.00	——	——
		Assessment exposure	Monitor stations (n=5)	1.00	0.98	1.03	80.3	＜0.001
			Models (n=3)	1.03	0.98	1.08	29.9	0.240
		Sample Size	<1000 (n=4)	1.00	0.82	1.17	41.5	0.163
			≥1000 (n=4)	1.02	1.01	1.03	0.0	0.883
		Diagnostic criteria	CES-D (n=2)	0.99	0.99	1.00	0.0	0.336
			EPDS (n=4)	1.00	0.83	1.17	41.4	0.163
			Others (n=2)	1.02	1.00	1.03	0.0	0.774
		Study quality	High (n=5)	1.02	1.01	1.04	0.0	0.451
			Moderate (n=2)	0.96	0.85	1.08	37.0	0.208
			Low (n=1)	1.02	1.01	1.03	——	——
	First trimester	Region	America (n=3)	0.98	0.98	0.99	0.0	0.750
			Asia (n=3)	1.01	0.96	1.06	69.7	0.037
		Study design	Cohort study (n=5)	1.00	0.96	1.04	45.2	0.121
			Time-series study (n=1)	0.98	0.98	0.99	——	——
		Assessment exposure	Monitor stations (n=5)	1.00	0.96	1.04	45.6	0.118
			Models (n=1)	0.98	0.96	1.00	——	——
		Sample Size	<1000 (n=3)	0.98	0.98	0.99	0.0	0.750
			≥1000 (n=3)	1.01	0.96	1.06	69.7	0.037
		Diagnostic criteria	CES-D (n=2)	0.98	0.98	0.99	0.0	0.553
			EPDS (n=2)	1.04	0.83	1.24	56.5	0.129
			Others (n=2)	1.00	0.95	0.95	76.9	0.037
		Study quality	High (n=4)	1.01	0.96	1.05	57.1	0.072
			Moderate (n=2)	0.98	0.98	0.99	0.0	0.635
	Second trimester	Region	America (n=3)	0.99	0.84	1.15	54.2	0.113
			Asia (n=3)	1.01	0.99	1.02	0.0	0.818
		Study design	Cohort study (n=5)	1.00	0.98	1.03	15.0	0.319
			Time-series study (n=1)	1.01	1.00	1.02	——	——
		Assessment	Monitor stations (n=5)	1.01	0.98	1.03	17.4	0.304

		exposure	Models (n=1)	1.01	0.99	1.03	——	——
		Sample Size	<1000 (n=3)	0.99	0.84	1.15	54.2	0.113
			≥1000 (n=3)	1.01	0.99	1.02	0.0	0.818
		Diagnostic criteria	CES-D (n=2)	1.14	0.73	1.54	55.0	0.136
			EPDS (n=2)	0.94	0.79	1.08	10.0	0.292
			Others (n=2)	1.01	0.99	1.02	0.0	0.531
		Study quality	High (n=4)	1.01	0.99	1.03	0.0	0.449
			Moderate (n=2)	0.97	0.85	1.09	53.4	0.143
	Third trimester	Region	America (n=3)	0.98	0.98	0.99	0.0	0.869
			Asia (n=3)	0.99	0.93	1.04	78.9	0.009
		Study design	Cohort study (n=5)	0.99	0.94	1.03	60.9	0.037
			Time-series study (n=1)	0.99	0.98	0.99	——	——
		Assessment exposure	Monitor stations (n=5)	0.98	0.94	1.01	45.3	0.120
			Models (n=1)	1.02	1.00	1.04	——	——
		Sample Size	<1000 (n=3)	0.98	0.98	0.99	0.0	0.869
			≥1000 (n=3)	0.99	0.93	1.04	78.9	0.009
		Diagnostic criteria	CES-D (n=2)	0.98	0.98	0.99	0.0	0.873
			EPDS (n=2)	0.86	0.71	1.01	41.6	0.191
			Others (n=2)	1.02	1.00	1.03	0.0	0.323
		Study quality	High (n=4)	0.99	0.94	1.04	68.6	0.023
			Moderate (n=2)	0.98	0.98	0.99	0.0	0.614
	Postpartum 3-6 months	Region	Asia (n=1)	1.01	0.98	1.05	——	——
		Study design	Cohort study (n=1)	1.01	0.98	1.05	——	——
		Assessment exposure	Models (n=1)	1.01	0.98	1.05	——	——
		Sample Size	≥1000 (n=1)	1.01	0.98	1.05	——	——
		Diagnostic criteria	Others (n=1)	1.01	0.98	1.05	——	——
		Study quality	High (n=1)	1.01	0.98	1.05	——	——
PM10	Whole pregnancy	Region	America (n=3)	1.01	1.00	1.02	0.0	0.438
			Europe (n=1)	1.06	0.96	1.16	——	——
			Asia (n=1)	1.48	1.33	1.62	——	——
		Study design	Cohort study (n=4)	1.18	0.98	1.38	92.0	＜0.001
			Time-series study (n=1)	1.01	0.99	1.02	——	——
		Assessment exposure	Monitor stations (n=3)	1.23	0.84	1.62	95.0	＜0.001
			Models (n=2)	1.02	1.00	1.04	0.0	0.442
		Sample Size	<1000 (n=2)	1.01	0.99	1.02	0.0	0.444
			≥1000 (n=3)	1.17	0.96	1.39	94.6	＜0.001
		Diagnostic criteria	CES-D (n=2)	1.01	0.99	1.02	0.0	0.444
			EPDS (n=2)	1.26	0.86	1.67	95.3	＜0.001
			Others (n=1)	1.02	1.00	1.04	——	——

	Study quality	High (n=4)	1.18	0.98	1.38	92.0	＜0.001
		Moderate (n=1)	1.01	0.99	1.02	——	——
First trimester	Region	America (n=3)	0.98	0.97	1.00	34.5	0.217
		Asia (n=1)	1.29	1.20	1.39	——	——
	Study design	Cohort study (n=3)	1.06	0.8	1.33	94.8	＜0.001
		Time-series study (n=1)	0.97	0.95	0.99	——	——
	Assessment exposure	Monitor stations (n=3)	1.05	0.78	1.33	95.3	＜0.001
		Models (n=1)	0.99	0.98	1.00	——	——
	Sample Size	<1000 (n=2)	0.97	0.95	0.99	0.0	0.332
		≥1000 (n=2)	1.14	0.84	1.44	97.3	＜0.001
	Diagnostic criteria	CES-D (n=2)	0.97	0.95	0.99	0.0	0.332
		EPDS (n=1)	1.29	1.20	1.39	——	——
		Others (n=1)	0.99	0.98	1.00	——	——
	Study quality	High (n=3)	1.06	0.80	1.33	94.8	＜0.001
		Moderate (n=1)	0.97	0.95	0.99	——	——
Second trimester	Region	America (n=3)	1.04	0.99	1.08	92.5	＜0.001
		Asia (n=1)	1.30	1.20	1.39	——	——
	Study design	Cohort study (n=3)	1.19	0.92	1.45	94.5	＜0.001
		Time-series study (n=1)	1.06	1.05	1.07	——	——
	Assessment exposure	Monitor stations (n=3)	1.20	0.97	1.42	92.3	＜0.001
		Models (n=1)	1.01	1.00	1.02	——	——
	Sample Size	<1000 (n=2)	1.11	0.84	1.39	28.0	0.238
		≥1000 (n=2)	1.15	0.87	1.43	97.1	＜0.001
	Diagnostic criteria	CES-D (n=2)	1.11	0.84	1.39	28.0	0.238
		EPDS (n=1)	1.30	1.20	1.39	——	——
		Others (n=1)	1.01	1.00	1.02	——	——
	Study quality	High (n=3)	1.19	0.92	1.45	94.5	＜0.001
		Moderate (n=1)	1.06	1.05	1.07	——	——
Third trimester	Region	America (n=3)	1.01	0.98	1.03	71.2	0.031
		Asia (n=1)	1.24	1.13	1.34	——	——
	Study design	Cohort study (n=3)	1.11	0.92	1.30	87.2	＜0.001
		Time-series study (n=1)	0.99	0.98	1.01	——	——
	Assessment exposure	Monitor stations (n=3)	1.10	0.88	1.31	90.0	＜0.001
		Models (n=1)	1.02	1.00	1.03	——	——
	Sample Size	<1000 (n=2)	0.99	0.98	1.01	0.0	0.917
		≥1000 (n=2)	1.12	0.91	1.33	93.6	＜0.001
	Diagnostic criteria	CES-D (n=2)	0.99	0.98	1.01	0.0	0.917
		EPDS (n=1)	1.24	1.13	1.34	——	——
		Others (n=1)	1.02	1.00	1.03	——	——
	Study quality	High (n=3)	1.11	0.92	1.30	87.2	＜0.001
		Moderate (n=1)	0.99	0.98	1.01	——	——

NO_2	Whole pregnancy	Region	America (n=3)	0.99	0.95	1.02	68.8	0.040
			Europe (n=1)	0.98	0.92	1.04	——	——
			Asia (n=1)	1.52	1.27	1.77	——	——
		Study design	Cohort study (n=4)	1.09	0.95	1.22	85.9	＜0.001
			Time-series study (n=1)	1.00	1.00	1.00	——	——
		Assessment exposure	Monitor stations (n=3)	1.33	0.86	1.81	89.7	＜0.001
			Models (n=2)	0.97	0.94	1.00	0.0	0.784
		Sample Size	<1000 (n=2)	1.25	0.51	2.00	61.7	0.106
			≥1000 (n=3)	1.07	0.94	1.20	89.2	＜0.001
		Diagnostic criteria	CES-D (n=2)	1.25	0.51	2.00	61.7	0.106
			EPDS (n=2)	1.24	0.71	1.77	94.1	＜0.001
			Others (n=1)	0.97	0.94	1.00	——	——
		Study quality	High (n=4)	1.09	0.95	1.22	85.9	＜0.001
			Moderate (n=1)	1.00	1.00	1.00	——	——
	First trimester	Region	America (n=3)	0.99	0.97	1.00	40.4	0.187
			Asia (n=2)	1.08	0.92	1.23	83.6	0.013
		Study design	Cohort study (n=4)	1.01	0.99	1.03	57.2	0.072
			Time-series study (n=1)	0.98	0.98	0.99	——	——
		Assessment exposure	Monitor stations (n=4)	1.00	0.98	1.03	91.7	＜0.001
			Models (n=1)	1.00	0.98	1.02	——	——
		Sample Size	<1000 (n=2)	0.98	0.98	0.99	0.0	0.476
			≥1000 (n=3)	1.01	0.99	1.03	69.7	0.037
		Diagnostic criteria	CES-D (n=2)	0.98	0.98	0.99	0.0	0.476
			EPDS (n=1)	1.17	1.04	1.30	——	——
			Others (n=2)	1.01	1.00	1.01	0.0	0.517
		Study quality	High (n=4)	1.01	0.99	1.03	57.2	0.072
			Moderate (n=1)	0.98	0.98	0.99	——	——
	Second trimester	Region	America (n=3)	1.02	0.98	1.06	78.6	0.009
			Asia (n=2)	1.15	0.85	1.46	94.4	＜0.001
		Study design	Cohort study (n=4)	1.04	0.99	1.09	86.0	＜0.001
			Time-series study (n=1)	1.03	1.03	1.04	——	——
		Assessment exposure	Monitor stations (n=4)	1.03	1.00	1.06	94.5	＜0.001
			Models (n=1)	1.00	0.98	1.02	——	——
		Sample Size	<1000 (n=2)	1.45	0.34	2.55	69.5	0.070
			≥1000 (n=3)	1.03	0.98	1.08	88.9	＜0.001
		Diagnostic criteria	CES-D (n=2)	1.45	0.34	2.55	69.5	0.070
			EPDS (n=1)	1.32	1.17	1.46	——	——
			Others (n=2)	1.01	1.00	1.01	0.0	0.651
		Study quality	High (n=4)	1.04	0.99	1.09	86.0	
			Moderate (n=1)	1.03	1.03	1.04	——	——
	Third	Region	America (n=3)	0.98	0.97	0.99	0.0	0.879

	trimest		Asia (n=2)	1.16	0.83	1.49	92.2	＜0.001
	er	Study design	Cohort study (n=4)	1.01	0.97	1.05	83.8	＜0.001
			Time-series study (n=1)	0.99	0.97	1.00	——	——
		Assessment exposure	Monitor stations (n=4)	1.00	0.97	1.03	85.6	
			Models (n=1)	0.98	0.96	1.00	——	——
		Sample Size	<1000 (n=2)	0.98	0.97	1.00	0.0	0.764
			≥1000 (n=3)	1.01	0.97	1.05	89.1	＜0.001
		Diagnostic criteria	CES-D (n=2)	0.98	0.97	1.00	0.0	0.764
			EPDS (n=1)	1.34	1.16	1.52	——	——
			Others (n=2)	0.99	0.97	1.02	81.3	0.021
		Study quality	High (n=4)	1.01	0.97	1.05	83.8	＜0.001
			Moderate (n=1)	0.99	0.97	1.00	——	——
	Postpartum 3-6 months	Region	Asia (n=1)	1.01	1.00	1.01	——	——
		Study design	Cohort study (n=1)	1.01	1.00	1.01	——	——
		Assessment exposure	Models (n=1)	1.01	1.00	1.01	——	——
		Sample Size	≥1000 (n=1)	1.01	1.00	1.01	——	——
		Diagnostic criteria	Others (n=1)	1.01	1.00	1.01	——	——
		Study quality	High (n=1)	1.01	1.00	1.01	——	——
O_3	Whole pregnancy	Region	America (n=3)	1.04	0.96	1.12	93.2	＜0.001
			Asia (n=1)	0.65	0.53	0.77	——	——
		Study design	Cohort study (n=3)	0.88	0.52	1.24	95.8	＜0.001
			Time-series study (n=1)	1.01	1.00	1.01	——	——
		Assessment exposure	Monitor stations (n=3)	0.85	0.55	1.14	94.0	＜0.001
			Models (n=1)	1.09	1.06	1.12	——	——
		Sample Size	<1000 (n=2)	1.01	1.00	1.01	0.0	0.634
			≥1000 (n=2)	0.88	0.45	1.30	97.9	＜0.001
		Diagnostic criteria	CES-D (n=2)	1.01	1.00	1.01	0.0	0.634
			EPDS (n=1)	0.65	0.53	0.77	——	——
			Others (n=1)	1.09	1.06	1.12	——	——
		Study quality	High (n=3)	0.88	0.52	1.24	95.8	＜0.001
			Moderate (n=1)	1.01	1.00	1.01	——	——
	First trimester	Region	America (n=3)	1.01	0.99	1.03	66.8	0.049
			Asia (n=1)	1.09	1.01	1.17	——	——
		Study design	Cohort study (n=3)	1.03	0.94	1.12	69.2	0.039
			Time-series study (n=1)	1.00	1.00	1.01	——	——
		Assessment exposure	Monitor stations (n=3)	1.02	0.92	1.12	75.2	0.018
			Models (n=1)	1.02	1.00	1.04	——	——
		Sample Size	<1000 (n=2)	0.88	0.56	1.21	67.2	0.081
			≥1000 (n=2)	1.05	0.98	1.12	67.3	0.080
		Diagnostic criteria	CES-D (n=2)	0.88	0.56	1.21	67.2	0.081
			EPDS (n=1)	1.09	1.01	1.17	——	——
			Others (n=1)	1.02	1.00	1.04	——	——

		Study quality	High (n=3)	1.03	0.94	1.12	69.2	0.039
			Moderate (n=1)	1.00	1.00	1.01	——	——
	Second trimester	Region	America (n=3)	1.01	1.00	1.02	52.0	0.124
			Asia (n=1)	0.76	0.69	0.83	——	——
		Study design	Cohort study (n=3)	0.87	0.64	1.10	96.5	＜0.001
			Time-series study (n=1)	1.01	1.00	1.01	——	——
		Assessment exposure	Monitor stations (n=3)	0.86	0.64	1.08	96.3	＜0.001
			Models (n=1)	1.02	1.00	1.03	——	——
		Sample Size	<1000 (n=2)	0.97	0.77	1.16	29.8	0.233
			≥1000 (n=2)	0.89	0.64	1.15	98.2	＜0.001
		Diagnostic criteria	CES-D (n=2)	0.97	0.77	1.16	29.8	0.233
			EPDS (n=1)	0.76	0.69	0.83	——	——
			Others (n=1)	1.02	1.00	1.03	——	——
		Study quality	High (n=3)	0.87	0.64	1.10	96.5	＜0.001
			Moderate (n=1)	1.01	1.00	1.01	——	——
	Third trimester	Region	America (n=3)	1.02	1.00	1.05	75.3	0.017
			Asia (n=1)	0.94	0.85	1.02	——	——
		Study design	Cohort study (n=3)	1.00	0.91	1.10	67.7	0.045
			Time-series study (n=1)	1.01	1.01	1.02	——	——
		Assessment exposure	Monitor stations (n=3)	0.99	0.92	1.06	49.4	0.139
			Models (n=1)	1.04	1.02	1.06	——	——
		Sample Size	<1000 (n=2)	1.01	0.95	1.08	1.4	0.314
			≥1000 (n=2)	1.00	0.90	1.10	81.1	0.021
		Diagnostic criteria	CES-D (n=2)	1.01	0.95	1.08	1.4	0.314
			EPDS (n=1)	0.94	0.85	1.02	——	——
			Others (n=1)	1.04	1.02	1.06	——	——
		Study quality	High (n=3)	1.00	0.91	1.10	67.7	0.045
			Moderate (n=1)	1.01	1.01	1.02	——	——
CO	Whole pregnancy	Region	Asia (n=1)	2.31	1.91	2.80	——	——
		Study design	Cohort study (n=1)	2.31	1.91	2.80	——	——
		Assessment exposure	Monitor stations (n=1)	2.31	1.91	2.80	——	——
		Sample Size	≥1000 (n=1)	2.31	1.91	2.80	——	——
		Diagnostic criteria	EPDS (n=1)	2.31	1.91	2.80	——	——
		Study quality	High (n=1)	2.31	1.91	2.80	——	——
	First trimester	Region	Asia (n=2)	1.25	0.77	1.73	95.6	＜0.001
		Study design	Cohort study (n=2)	1.25	0.77	1.73	95.6	＜0.001
		Assessment exposure	Monitor stations (n=2)	1.25	0.77	1.73	95.6	＜0.001
		Sample Size	≥1000 (n=2)	1.25	0.77	1.73	95.6	＜0.001
		Diagnostic criteria	EPDS (n=1)	1.50	1.32	1.72	——	——
			Others (n=1)	1.02	0.99	1.05	——	——

		Study quality	High (n=2)	1.25	0.77	1.73	95.6	＜0.001
	Second trimester	Region	Asia (n=2)	1.31	0.70	1.93	97.1	＜0.001
		Study design	Cohort study (n=2)	1.31	0.70	1.93	97.1	＜0.001
		Assessment exposure	Monitor stations (n=2)	1.31	0.70	1.93	97.1	＜0.001
		Sample Size	≥1000 (n=2)	1.31	0.70	1.93	97.1	＜0.001
		Diagnostic criteria	EPDS (n=1)	1.63	1.44	1.85	——	——
			Others (n=1)	1.01	0.98	1.04	——	——
		Study quality	High (n=2)	1.31	0.70	1.93	97.1	＜0.001
	Third trimester	Region	Asia (n=2)	1.10	0.88	1.32	83.3	0.014
		Study design	Cohort study (n=2)	1.10	0.88	1.32	83.3	0.014
		Assessment exposure	Monitor stations (n=2)	1.10	0.88	1.32	83.3	0.014
		Sample Size	≥1000 (n=2)	1.10	0.88	1.32	83.3	0.014
		Diagnostic criteria	EPDS (n=1)	1.23	1.06	1.42	——	——
			Others (n=1)	1.00	0.97	1.03	——	——
		Study quality	High (n=2)	1.10	0.88	1.32	83.3	0.014
	Postpartum 3-6 months	Region	Asia (n=1)	1.01	0.98	1.04	——	——
		Study design	Cohort study (n=1)	1.01	0.98	1.04	——	——
		Assessment exposure	Monitor stations (n=1)	1.01	0.98	1.04	——	——
		Sample Size	≥1000 (n=1)	1.01	0.98	1.04	——	——
		Diagnostic criteria	Others (n=1)	1.01	0.98	1.04	——	——
		Study quality	High (n=1)	1.01	0.98	1.04	——	——
SO_2	Whole pregnancy	Region	Asia (n=1)	0.98	0.89	1.09	——	——
		Study design	Cohort study (n=1)	0.98	0.89	1.08	——	——
		Assessment exposure	Monitor stations (n=1)	0.98	0.89	1.08	——	——
		Sample Size	≥1000 (n=1)	0.98	0.89	1.08	——	——
		Diagnostic criteria	EPDS (n=1)	0.98	0.89	1.08	——	——
		Study quality	High (n=1)	0.98	0.89	1.08	——	——
	First trimester	Region	Asia (n=1)	0.96	0.88	1.06	——	——
		Study design	Cohort study (n=1)	0.96	0.88	1.06	——	——
		Assessment exposure	Monitor stations (n=1)	0.96	0.88	1.06	——	——
		Sample Size	≥1000 (n=1)	0.96	0.88	1.06	——	——
		Diagnostic criteria	EPDS (n=1)	0.96	0.88	1.06	——	——
		Study quality	High (n=1)	0.96	0.88	1.06	——	——

	Second trimester	Region	Asia (n=1)	1.03	0.95	1.13	——	——
		Study design	Cohort study (n=1)	1.03	0.95	1.13	——	——
		Assessment exposure	Monitor stations (n=1)	1.03	0.95	1.13	——	——
		Sample Size	≥1000 (n=1)	1.03	0.95	1.13	——	——
		Diagnostic criteria	EPDS (n=1)	1.03	0.95	1.13	——	——
		Study quality	High (n=1)	1.03	0.95	1.13	——	——
	Third trimester	Region	Asia (n=1)	0.96	0.89	1.03	——	——
		Study design	Cohort study (n=1)	0.96	0.89	1.03	——	——
		Assessment exposure	Monitor stations (n=1)	0.96	0.89	1.03	——	——
		Sample Size	≥1000 (n=1)	0.96	0.89	1.03	——	——
		Diagnostic criteria	EPDS (n=1)	0.96	0.89	1.03	——	——
		Study quality	High (n=1)	0.96	0.89	1.03	——	——
PM2.5 BC	Whole pregnancy	Region	America (n=1)	1.04	1.00	1.09	——	——
		Study design	Cohort study (n=1)	1.04	1.00	1.09	——	——
		Assessment exposure	Models (n=1)	1.04	1.00	1.09	——	——
		Sample Size	≥1000 (n=1)	1.04	1.00	1.09	——	——
		Diagnostic criteria	Others (n=1)	1.04	1.00	1.09	——	——
		Study quality	High (n=1)	1.04	1.00	1.09	——	——
	First trimester	Region	America (n=1)	1.03	1.00	1.05	——	——
		Study design	Cohort study (n=1)	1.03	1.00	1.05	——	——
		Assessment exposure	Models (n=1)	1.03	1.00	1.05	——	——
		Sample Size	≥1000 (n=1)	1.03	1.00	1.05	——	——
		Diagnostic criteria	Others (n=1)	1.03	1.00	1.05	——	——
		Study quality	High (n=1)	1.03	1.00	1.05	——	——
	Second trimester	Region	America (n=1)	0.99	0.97	1.03	——	——
		Study design	Cohort study (n=1)	0.99	0.97	1.03	——	——
		Assessment exposure	Models (n=1)	0.99	0.97	1.03	——	——
		Sample Size	≥1000 (n=1)	0.99	0.97	1.03	——	——
		Diagnostic criteria	Others (n=1)	0.99	0.97	1.03	——	——
		Study quality	High (n=1)	0.99	0.97	1.03	——	——
	Third trimester	Region	America (n=1)	1.01	0.99	1.05	——	——
		Study design	Cohort study (n=1)	1.01	0.99	1.05	——	——
		Assessment	Models (n=1)	1.01	0.99	1.05	——	——

		exposure						
		Sample Size	≥1000 (n=1)	1.01	0.99	1.05	——	——
		Diagnostic criteria	Others (n=1)	1.01	0.99	1.05	——	——
		Study quality	High (n=1)	1.01	0.99	1.05	——	——
PM2.5 SO_4^{2-}	Whole pregnancy	Region	America (n=1)	1.04	1.00	1.09	——	——
		Study design	Cohort study (n=1)	1.04	1.00	1.09	——	——
		Assessment exposure	Models (n=1)	1.04	1.00	1.09	——	——
		Sample Size	≥1000 (n=1)	1.04	1.00	1.09	——	——
		Diagnostic criteria	Others (n=1)	1.04	1.00	1.09	——	——
		Study quality	High (n=1)	1.04	1.00	1.09	——	——
	First trimester	Region	America (n=1)	1.03	1.00	1.05	——	——
		Study design	Cohort study (n=1)	1.03	1.00	1.05	——	——
		Assessment exposure	Models (n=1)	1.03	1.00	1.05	——	——
		Sample Size	≥1000 (n=1)	1.03	1.00	1.05	——	——
		Diagnostic criteria	Others (n=1)	1.03	1.00	1.05	——	——
		Study quality	High (n=1)	1.03	1.00	1.05	——	——
	Second trimester	Region	America (n=1)	0.99	0.97	1.03	——	——
		Study design	Cohort study (n=1)	0.99	0.97	1.03	——	——
		Assessment exposure	Models (n=1)	0.99	0.97	1.03	——	——
		Sample Size	≥1000 (n=1)	0.99	0.97	1.03	——	——
		Diagnostic criteria	Others (n=1)	0.99	0.97	1.03	——	——
		Study quality	High (n=1)	0.99	0.97	1.03	——	——
	Third trimester	Region	America (n=1)	1.01	0.99	1.05	——	——
		Study design	Cohort study (n=1)	1.01	0.99	1.05	——	——
		Assessment exposure	Models (n=1)	1.01	0.99	1.05	——	——
		Sample Size	≥1000 (n=1)	1.01	0.99	1.05	——	——
		Diagnostic criteria	Others (n=1)	1.01	0.99	1.05	——	——
		Study quality	High (n=1)	1.01	0.99	1.05	——	——
PM2.5 NO_3^-	Whole pregnancy	Region	America (n=1)	1.01	0.99	1.04	——	——
		Study design	Cohort study (n=1)	1.01	0.99	1.04	——	——
		Assessment exposure	Models (n=1)	1.01	0.99	1.04	——	——
		Sample Size	≥1000 (n=1)	1.01	0.99	1.04	——	——

		Diagnostic criteria	Others (n=1)	1.01	0.99	1.04	——	——
		Study quality	High (n=1)	1.01	0.99	1.04	——	——
	First trimester	Region	America (n=1)	0.99	0.97	1.00	——	——
		Study design	Cohort study (n=1)	0.99	0.97	1.00	——	——
		Assessment exposure	Models (n=1)	0.99	0.97	1.00	——	——
		Sample Size	≥1000 (n=1)	0.99	0.97	1.00	——	——
		Diagnostic criteria	Others (n=1)	0.99	0.97	1.00	——	——
		Study quality	High (n=1)	0.99	0.97	1.00	——	——
	Second trimester	Region	America (n=1)	1.02	1.01	1.04	——	——
		Study design	Cohort study (n=1)	1.02	1.01	1.04	——	——
		Assessment exposure	Models (n=1)	1.02	1.01	1.04	——	——
		Sample Size	≥1000 (n=1)	1.02	1.01	1.04	——	——
		Diagnostic criteria	Others (n=1)	1.02	1.01	1.04	——	——
		Study quality	High (n=1)	1.02	1.01	1.04	——	——
	Third trimester	Region	America (n=1)	1.00	0.99	1.02	——	——
		Study design	Cohort study (n=1)	1.00	0.99	1.02	——	——
		Assessment exposure	Models (n=1)	1.00	0.99	1.02	——	——
		Sample Size	≥1000 (n=1)	1.00	0.99	1.02	——	——
		Diagnostic criteria	Others (n=1)	1.00	0.99	1.02	——	——
		Study quality	High (n=1)	1.00	0.99	1.02	——	——
PM2.5 NH_4^+	Whole pregnancy	Region	America (n=1)	1.02	0.99	1.04	——	——
		Study design	Cohort study (n=1)	1.02	0.99	1.04	——	——
		Assessment exposure	Models (n=1)	1.02	0.99	1.04	——	——
		Sample Size	≥1000 (n=1)	1.02	0.99	1.04	——	——
		Diagnostic criteria	Others (n=1)	1.02	0.99	1.04	——	——
		Study quality	High (n=1)	1.02	0.99	1.04	——	——
	First trimester	Region	America (n=1)	0.99	0.97	1.00	——	——
		Study design	Cohort study (n=1)	0.99	0.97	1.00	——	——
		Assessment exposure	Models (n=1)	0.99	0.97	1.00	——	——
		Sample Size	≥1000 (n=1)	0.99	0.97	1.00	——	——
		Diagnostic criteria	Others (n=1)	0.99	0.97	1.00	——	——

	Study quality	High (n=1)	0.99	0.97	1.00	——	——
Second trimester	Region	America (n=1)	1.02	1.01	1.03	——	——
	Study design	Cohort study (n=1)	1.02	1.01	1.03	——	——
	Assessment exposure	Models (n=1)	1.02	1.01	1.03	——	——
	Sample Size	≥1000 (n=1)	1.02	1.01	1.03	——	——
	Diagnostic criteria	Others (n=1)	1.02	1.01	1.03	——	——
	Study quality	High (n=1)	1.02	1.01	1.03	——	——
Third trimester	Region	America (n=1)	1.01	0.99	1.02	——	——
	Study design	Cohort study (n=1)	1.01	0.99	1.02	——	——
	Assessment exposure	Models (n=1)	1.01	0.99	1.02	——	——
	Sample Size	≥1000 (n=1)	1.01	0.99	1.02	——	——
	Diagnostic criteria	Others (n=1)	1.01	0.99	1.02	——	——
	Study quality	High (n=1)	1.01	0.99	1.02	——	——

Table S7. The meta-regression to assess publication bias.

Type of air pollutants	Subgroup	p value
$PM_{2.5}$	Exposure window	0.592
	Region	0.599
	Study design	**0.002**
	Assessment exposure	0.355
	Sample Size	**0.002**
	Diagnostic criteria	**0.003**
	Study quality	0.743
PM_{10}	Exposure window	0.839
	Region	0.050
	Study design	0.530
	Assessment exposure	0.420
	Sample Size	0.920
	Diagnostic criteria	0.961
	Study quality	0.530
NO_2	Exposure window	0.904
	Region	0.238
	Study design	0.621
	Assessment exposure	0.296
	Sample Size	0.318
	Diagnostic criteria	0.688
	Study quality	0.621
O_3	Exposure window	0.940

Region	0.051
Study design	0.701
Assessment exposure	0.415
Sample Size	0.752
Diagnostic criteria	0.831
Study quality	0.701

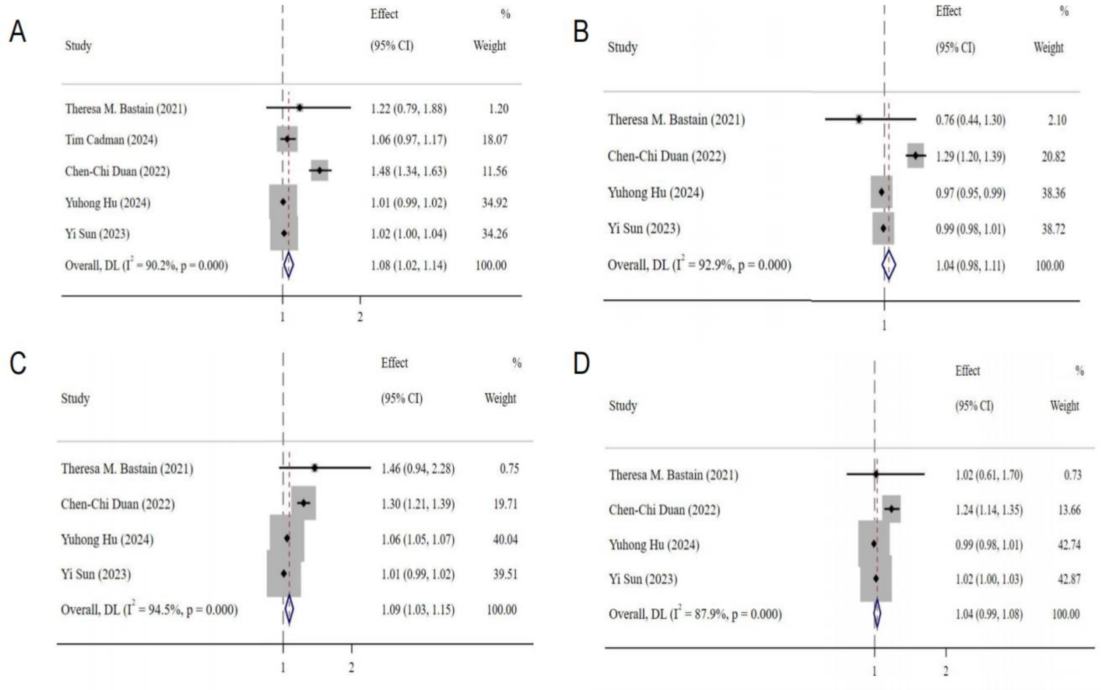

Figure S1. Meta-analysis of PM_{10} exposure and the risk of PPD. (A) the pregnancy average, (B) first trimester, (C) second trimester, (D) third trimester.

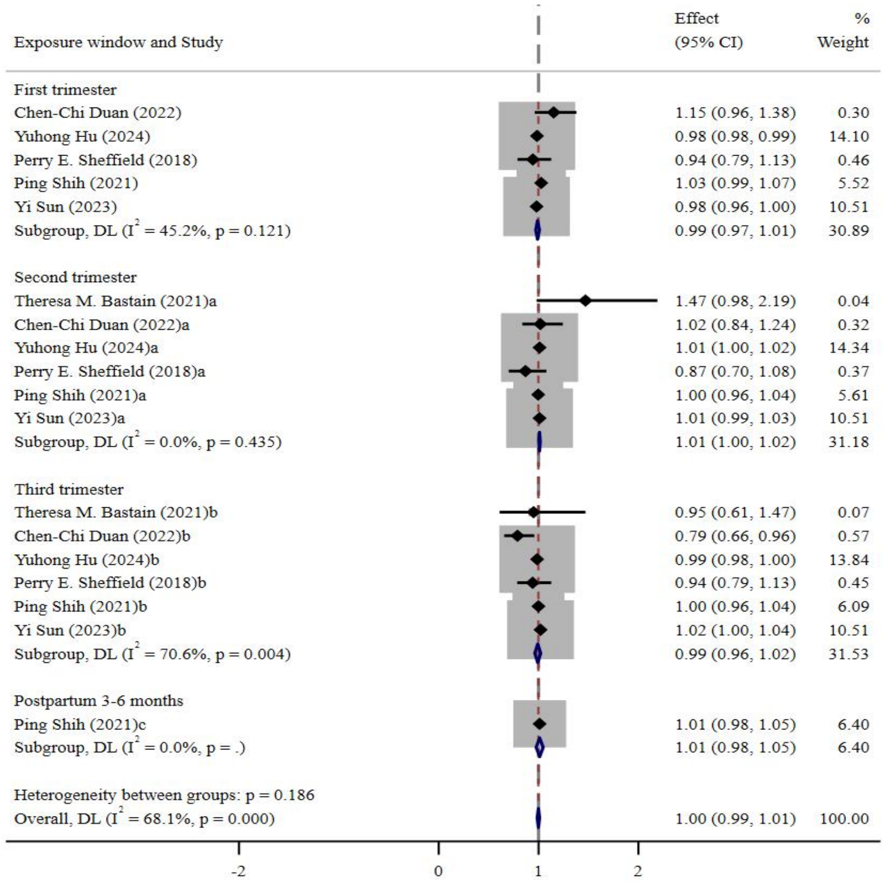

Figure S2. Meta-analysis of $PM_{2.5}$ exposure and the risk of PPD.

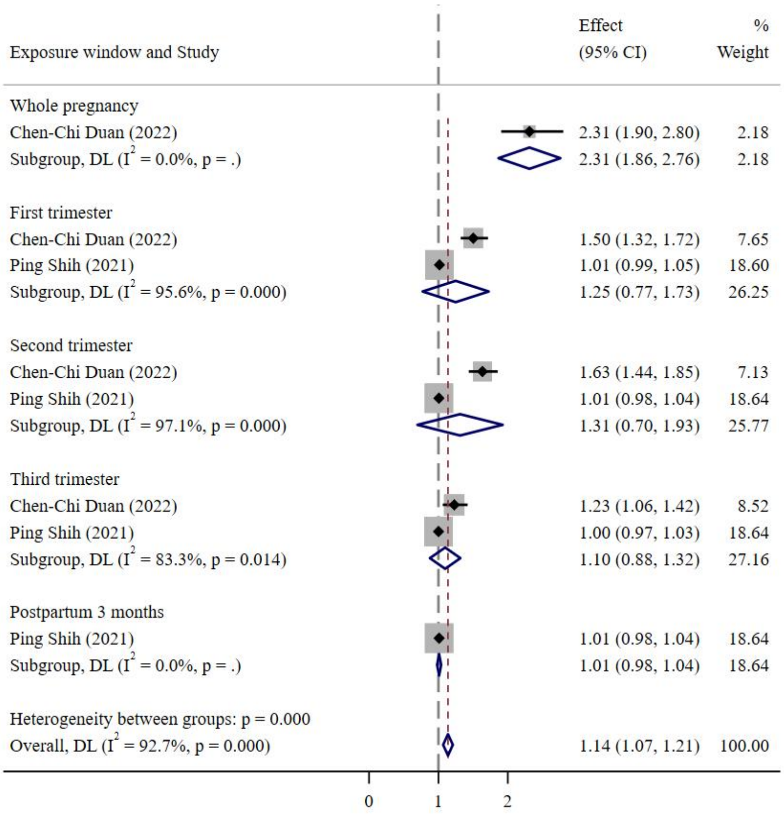

Figure S3. Meta-analysis of CO exposure and the risk of PPD.

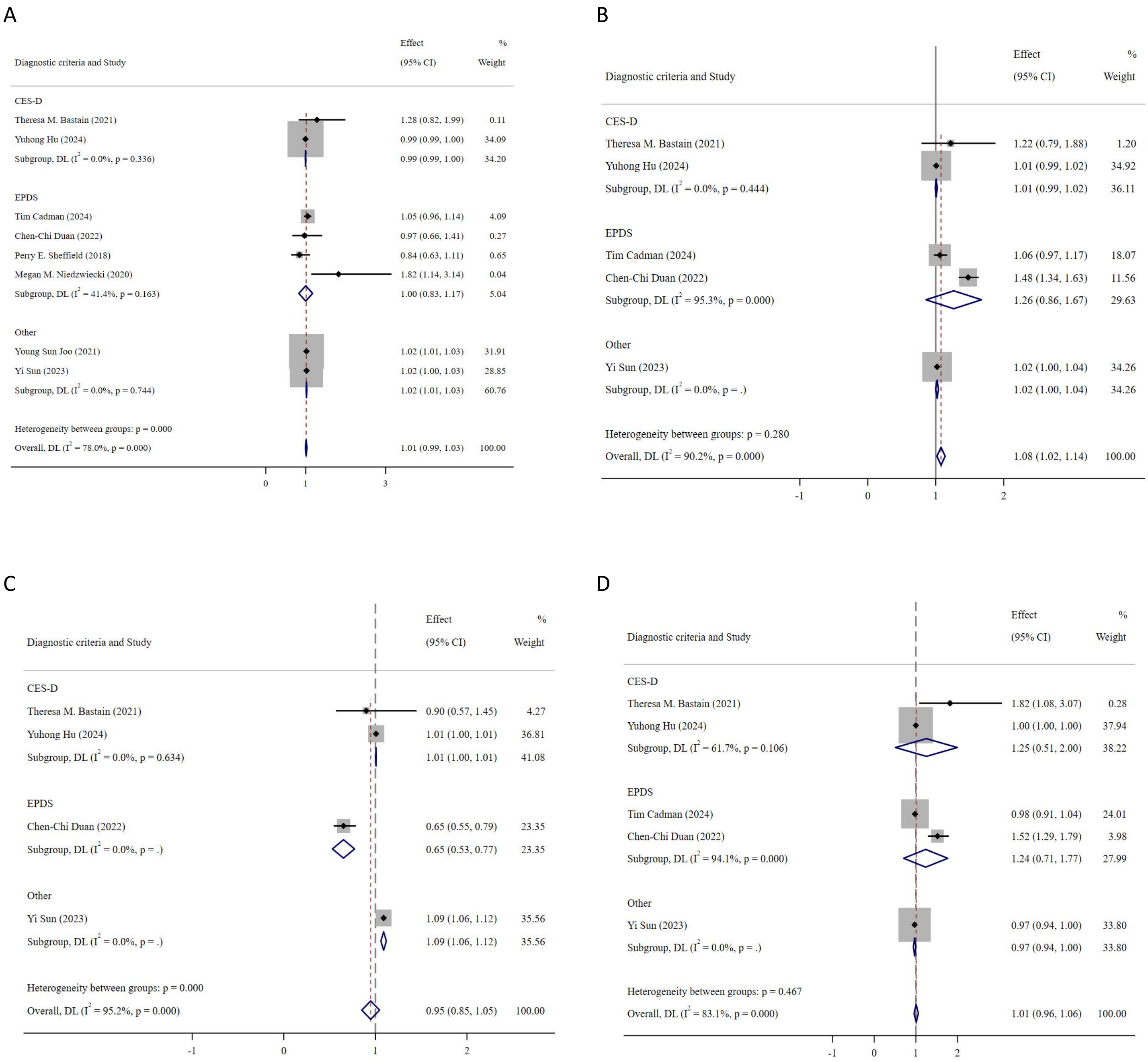

Figure S4. Stratified analysis of defined (CES-D, EPDS, ICD, etc.) with PPD. (A) $PM_{2.5}$, (B) PM_{10}, (C) O_3, (D) NO_2.

A

B

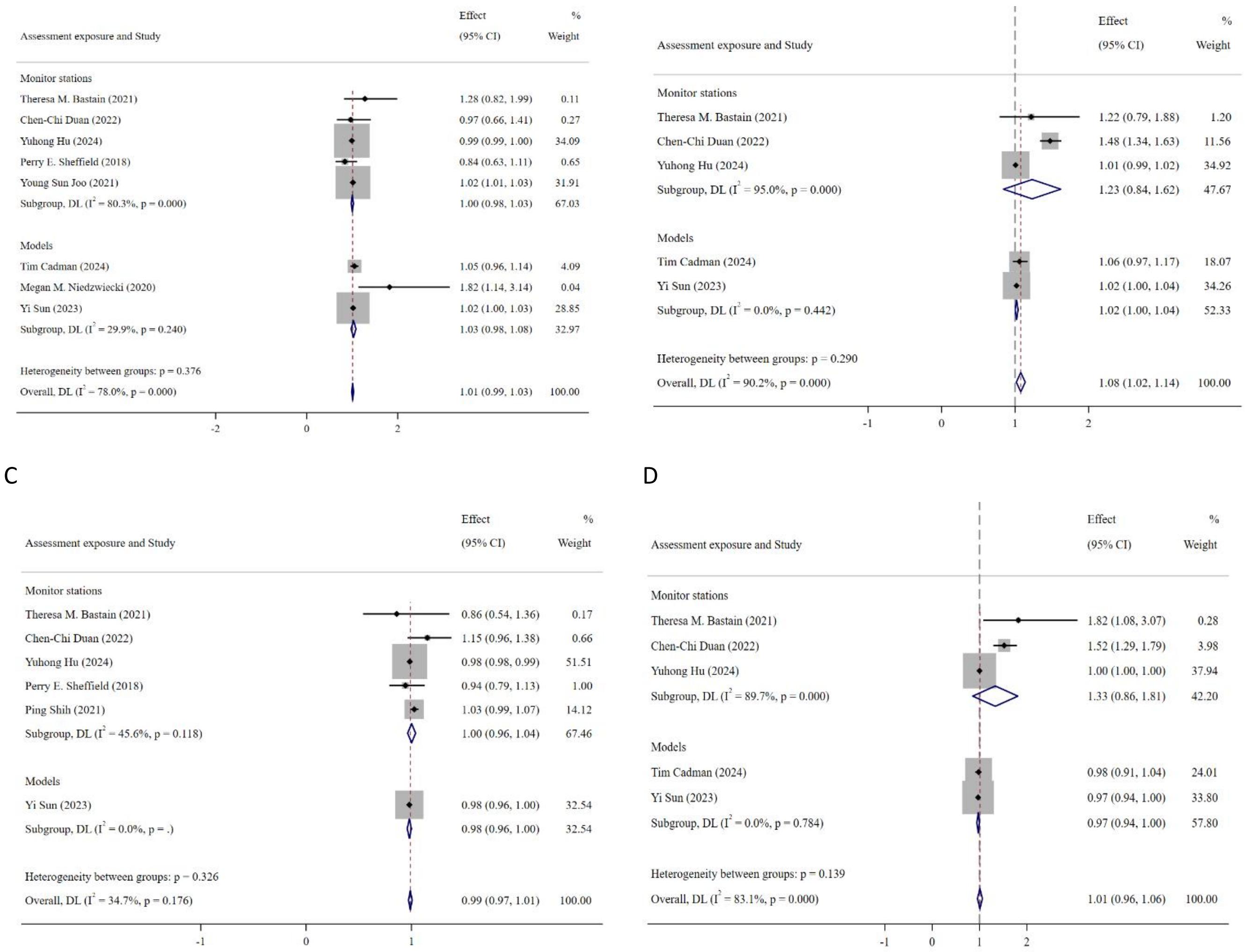

Figure S5. Stratified analysis of exposure assessment methods (e.g., satellite, models) with PPD. (A) $PM_{2.5}$, (B) PM_{10}, (C) O_3, (D) NO_2.

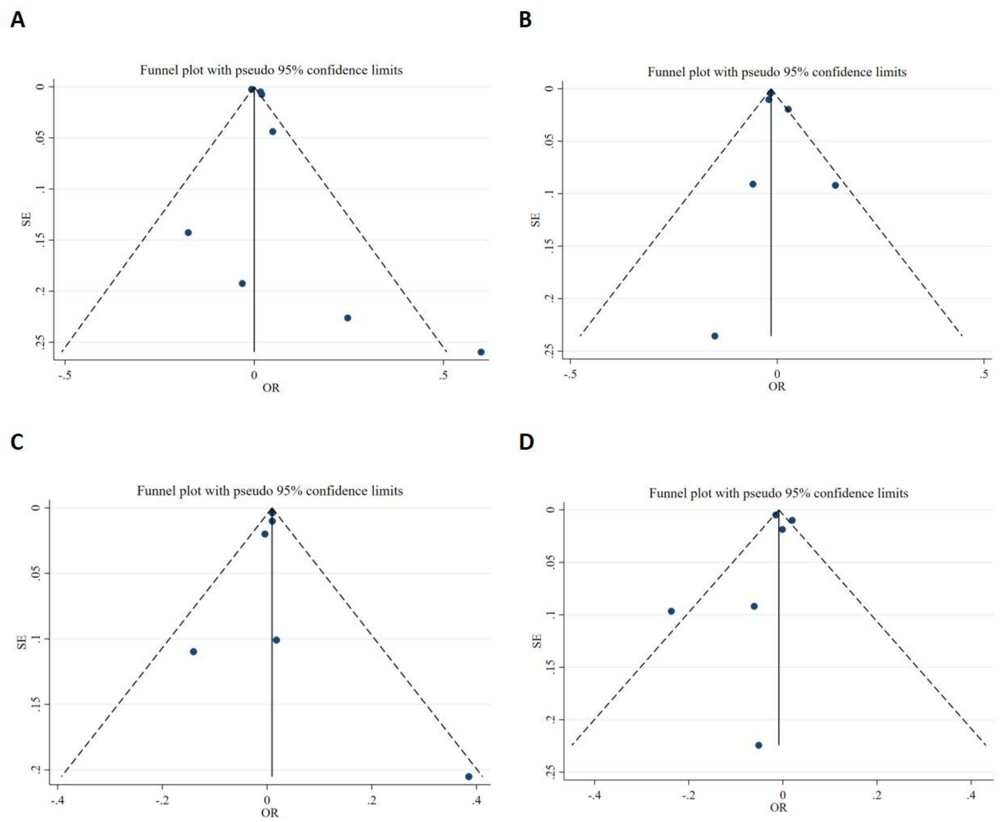

Figure S4. Funnel plots of the combined associations of $PM_{2.5}$ with PPD stratified by the whole pregnancy (A), first trimester (B), second trimester (C), or third trimester (D).

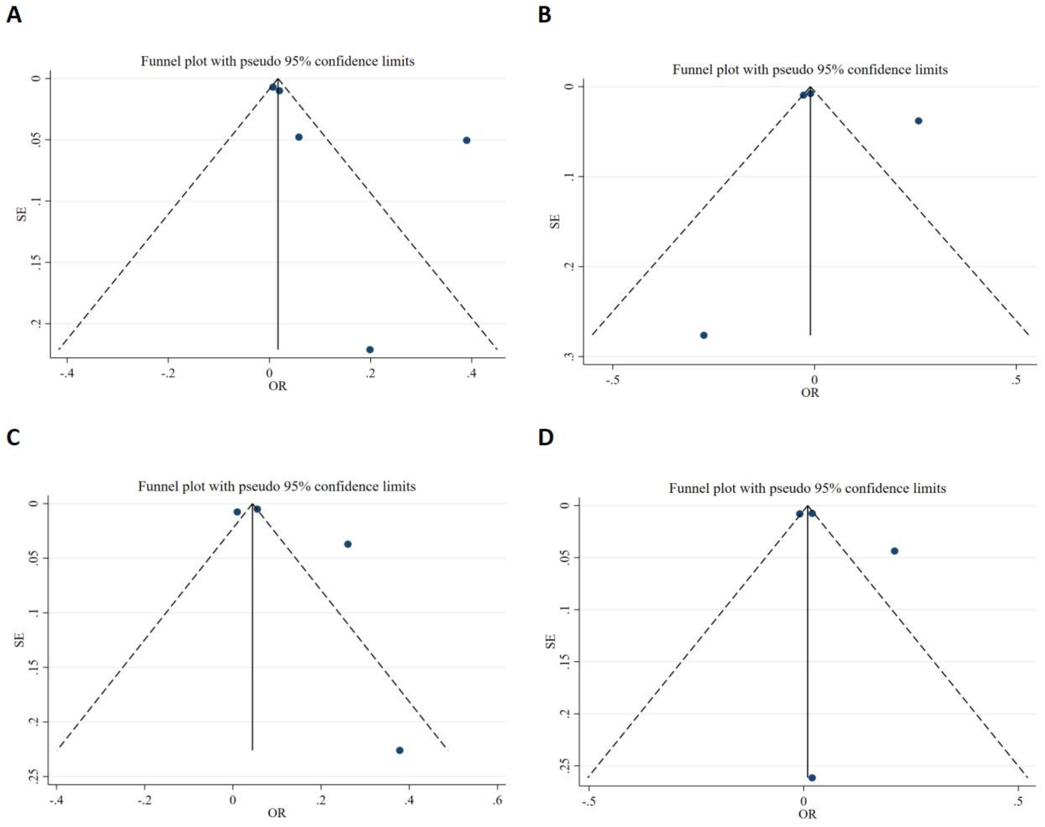

Figure S5. Funnel plots of the combined associations of PM_{10} with PPD stratified by the whole pregnancy (A), first trimester (B), second trimester (C), or third trimester (D).

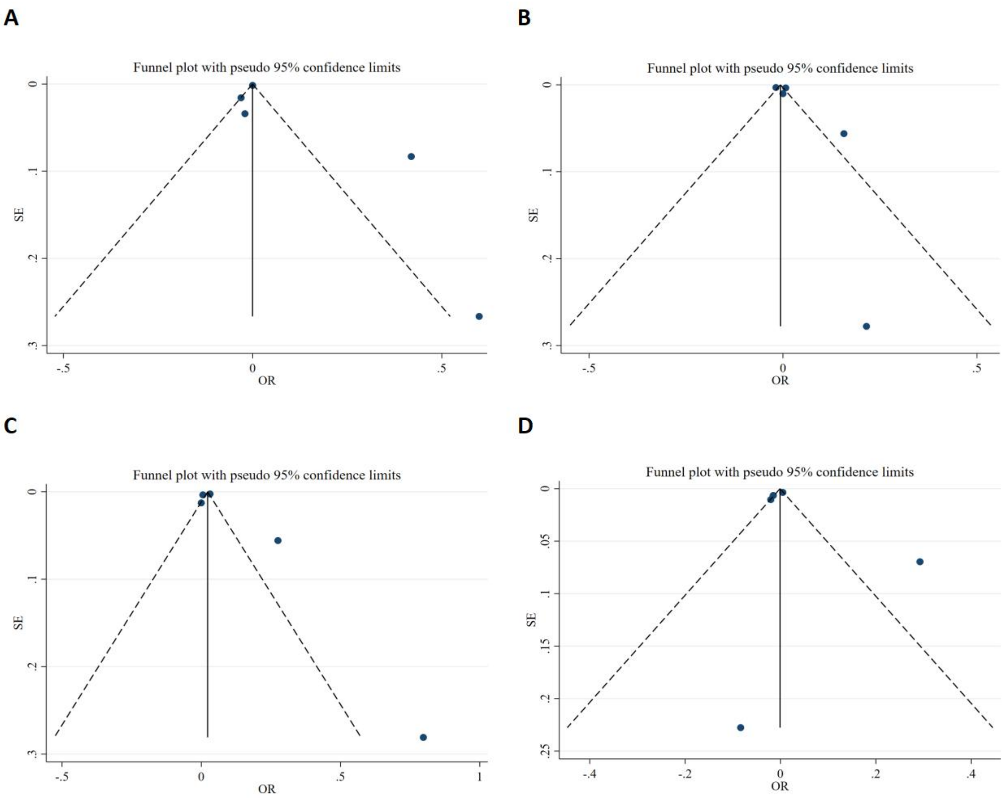

Figure S6. Funnel plots of the combined associations of NO_2 with PPD stratified by the whole pregnancy (A), first trimester (B), second trimester (C), or third trimester (D).

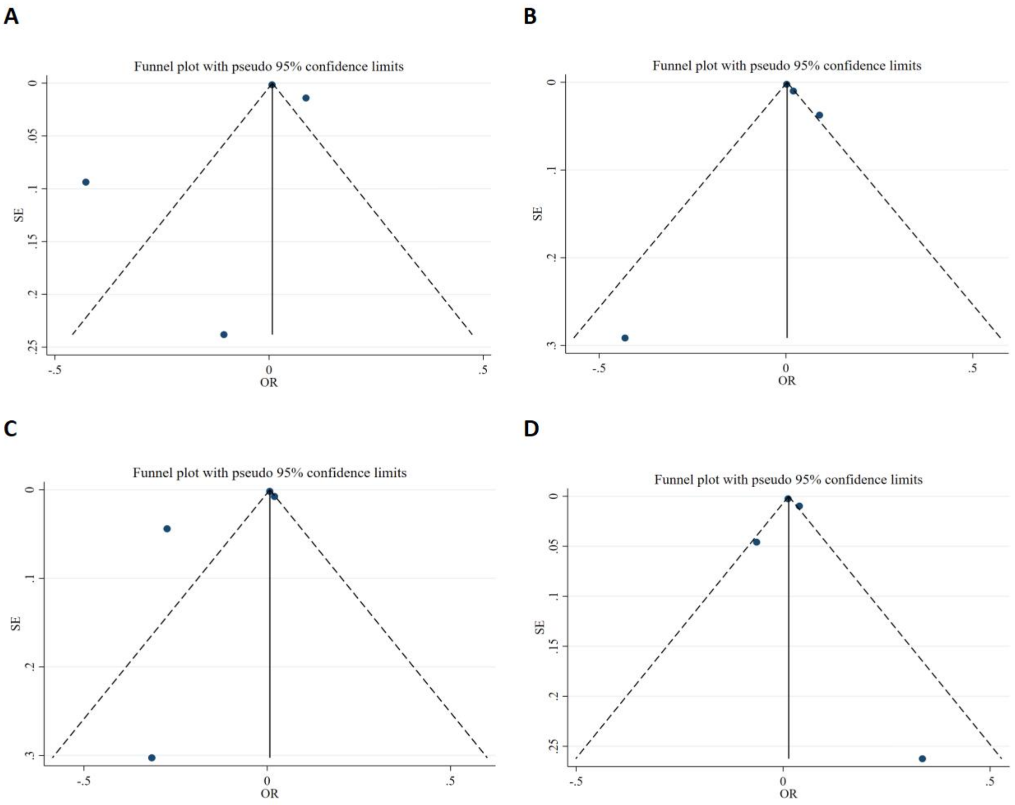

Figure S7. Funnel plots of the combined associations of O_3 with PPD stratified by the whole pregnancy (A), first trimester (B), second trimester (C), or third trimester (D).

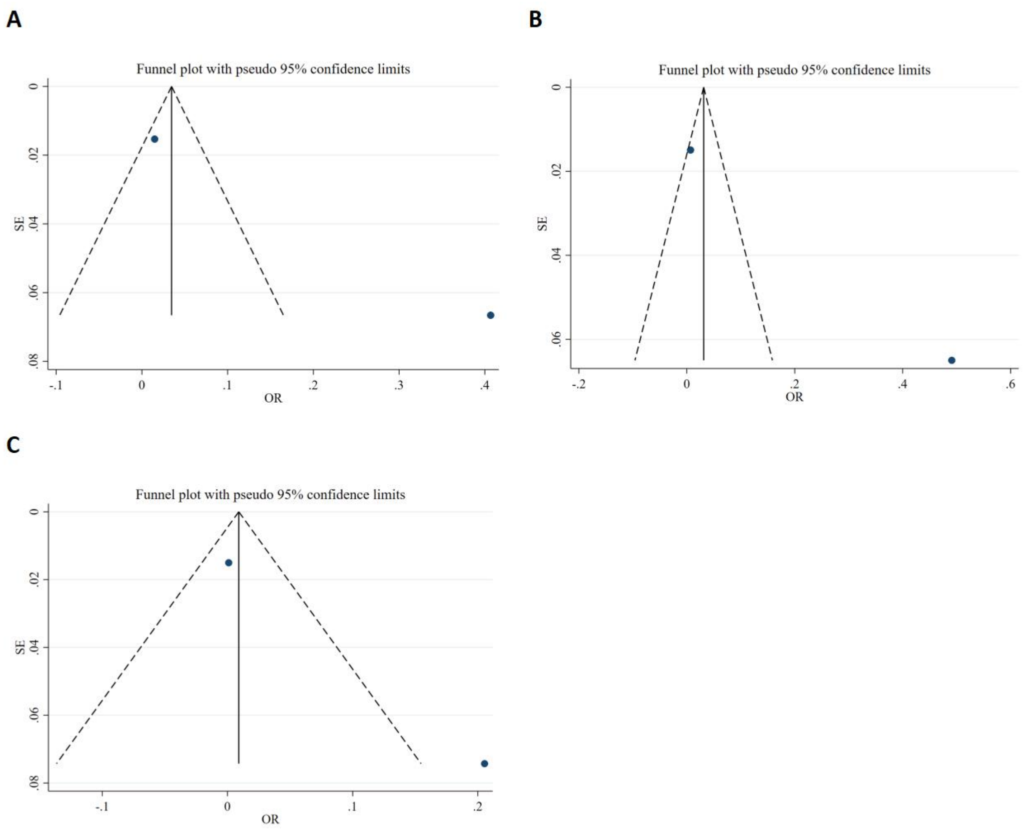

Figure S8. Funnel plots of the combined associations of CO with PPD stratified by the first trimester (A), second trimester (B), or third trimester (C).

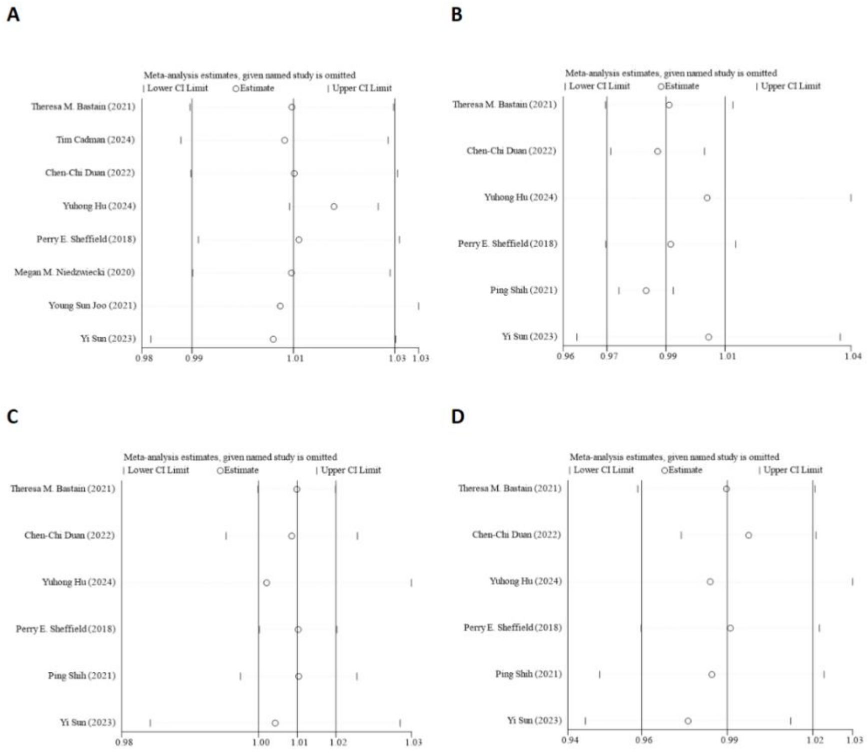

Figure S9. Leave-one-out sensitivity analysis to assess the association between PM2.5 and PPD stratified by the whole pregnancy (A), first trimester (B), second trimester (C), or third trimester (D).

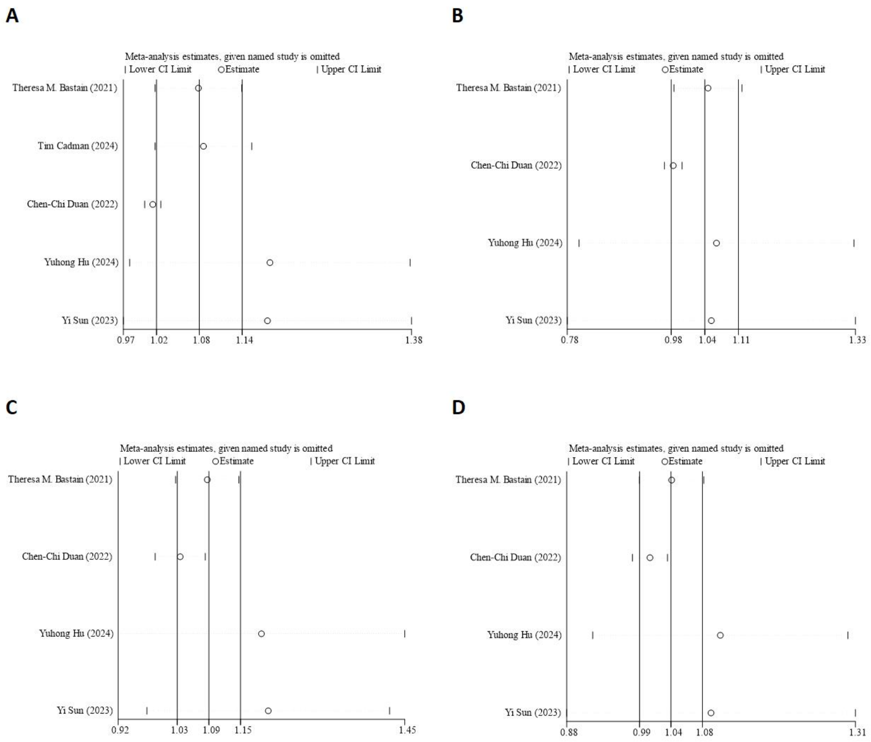

Figure S10. Leave-one-out sensitivity analysis to assess the association between PM10 and PPD stratified by the whole pregnancy (A), first trimester (B), second trimester (C), or third trimester (D).

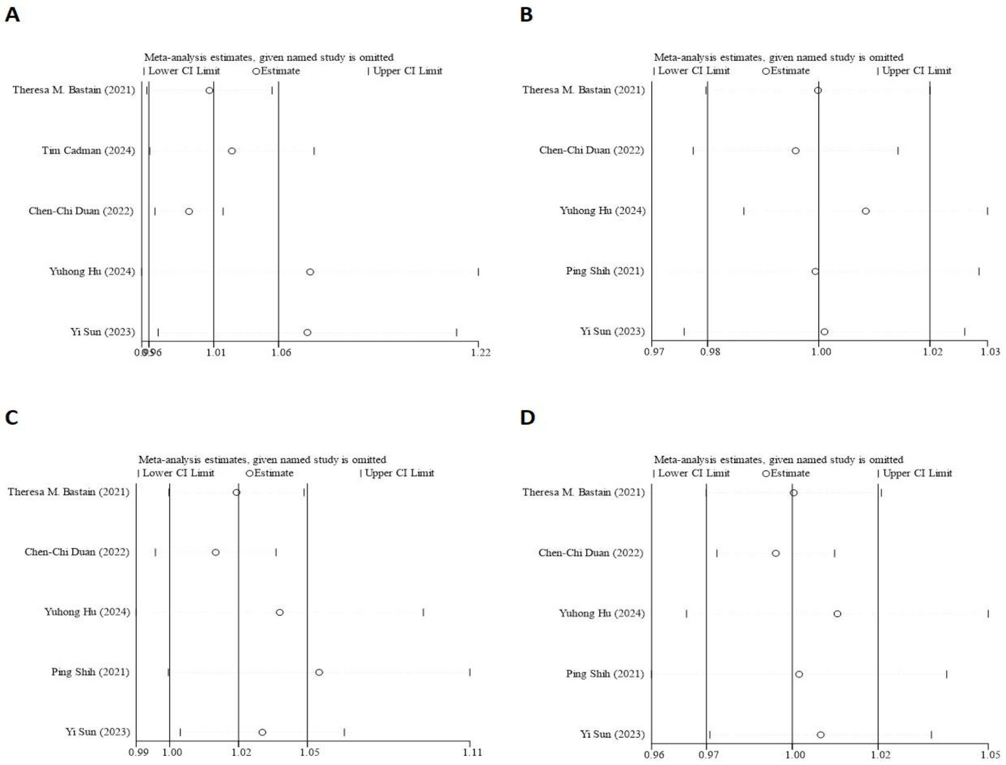

Figure S11. Leave-one-out sensitivity analysis to assess the association between NO_2 and PPD stratified by the whole pregnancy (A), first trimester (B), second trimester (C), or third trimester (D).

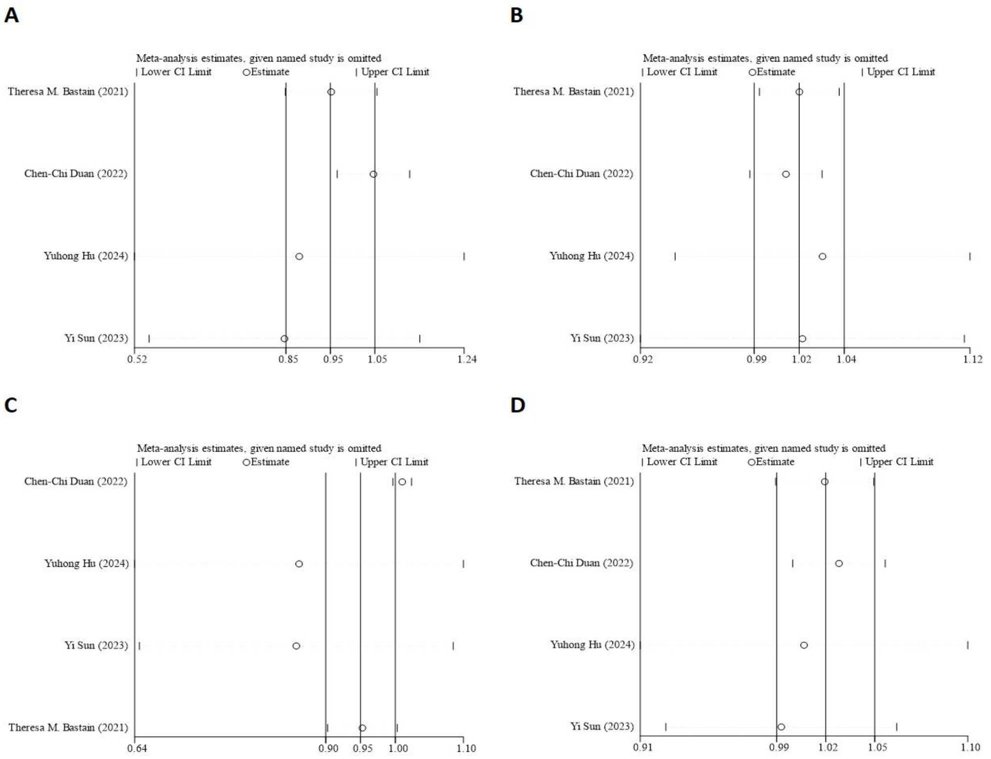

Figure S12. Leave-one-out sensitivity analysis to assess the association between O_3 and PPD stratified by the whole pregnancy (A), first trimester (B), second trimester (C), or third trimester (D).

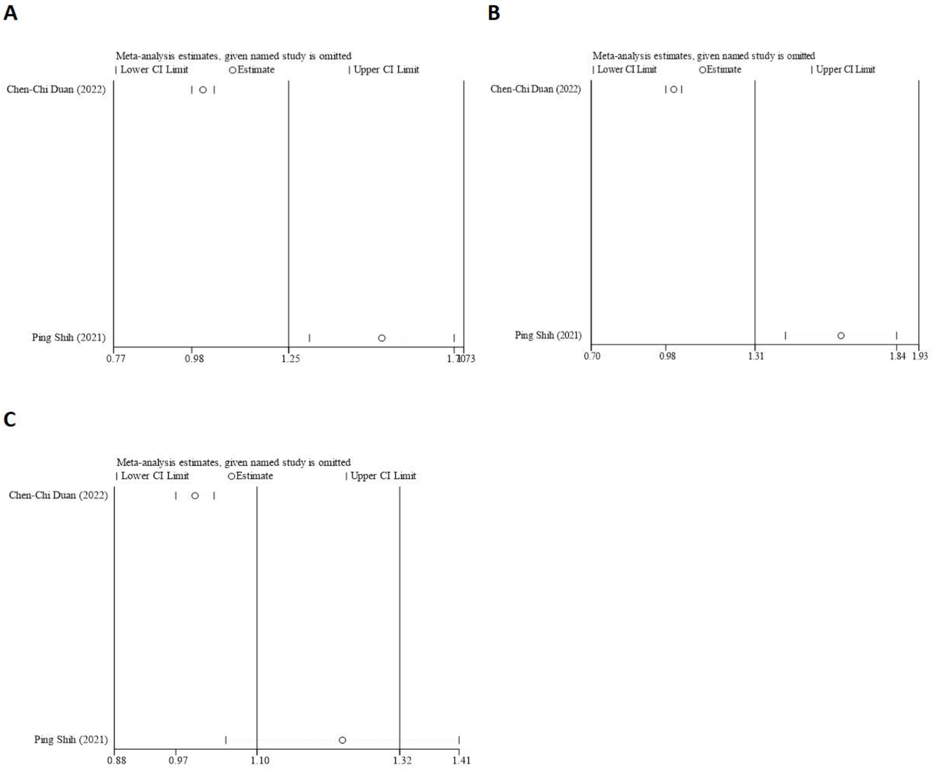

Figure S13. Leave-one-out sensitivity analysis to assess the association between CO and PPD stratified by the first trimester (A), second trimester (B), or third trimester (C).
